# Distinguishing Pedohebephebophilic Actors and Non-Actors: A Meta-Analysis

**DOI:** 10.1177/10790632251389171

**Published:** 2025-11-10

**Authors:** Agatha Chronos, Sara Jahnke

**Affiliations:** 1 Department of Health Promotion and Development, 1658 University of Bergen, Bergen, Norway

**Keywords:** Pedohebephebophilia, child sexual abuse, child sexual abuse material, meta-analysis, distinguishing factors

## Abstract

Distinguishing factors between pedohebephebophilic actors and non-actors remain a critical area of research for understanding offending behavior and developing targeted interventions. This meta-analysis synthesizes evidence on motivating, facilitating, situational, and other factors that differentiate individuals who have committed sexual offenses against children from those who have not. Following PRISMA guidelines, systematic searches were conducted across PsycNet, ProQuest, Web of Science, PubMed, and PSYNDEX, supplemented by manual searches. Data were analyzed using fixed and random effects models. From 2,185 records screened, 34 studies from 22 datasets met inclusion criteria. We conducted meta-analyses for 50 potential distinguishing factors. The strongest effect sizes were discovered for intelligence (*g* = -.86), stigma (*g* = .61), male sex (*g* = .51), age (*g* = .48), therapy attendance (*g* = .43) and interest (*g* = .43), and sexual (g = .38) and non-sexual (g = .38) adverse childhood experiences. The average quality score was 11.13 (*SD* = 1.82) out of maximum score of 16. Findings provide support for some motivating, facilitating, situational, and other factors distinguishing pedohebephilic actors from non-actors. These findings offer opportunities for improved risk assessment, prevention strategies, and therapeutic interventions, however, they are limited by the cross-sectional nature of the results.

## Introduction

Sexual interest in children includes nepiophilia, the attraction to infants and toddlers; pedophilia, the attraction to prepubescent children; hebephilia, the attraction to pubescent children; and ephebophilia, the attraction to postpubescent adolescents (Seto, 2017). Due to its potential to motivate child sexual abuse (CSA) (Dombert et al., 2016), sexual interest in children has received much public and scientific attention (Abel et al., 1984, 1989; Barbaree & Seto, 1997; Bernard, 1975; Drapeau et al., 2005; Jahnke et al., 2022; Seto, 2012). This has contributed to a large body of research, including meta-analyses, on risk factors for recidivism among people who have sexually offended (Hanson & Morton-Bourgon, 2019; Helmus et al., 2013). Recently, research and clinical interests have shifted towards people with a sexual interest in children from community settings (Beier et al., 2015; Cacciatori, 2017; Dymond & Duff, 2020; Jimenez-Arista & Reid, 2022; Walker, 2017). Initiatives such as Stop It Now! (n.d.) and *Prevention Project Dunkelfeld* (Stephens et al., 2022) offer treatment services and/or support for people with a sexual interest in children from the community, who are concerned about being at risk of committing sexual offenses. It is unclear if risk factors found in the forensic literature can be applied to a non-forensic population comprised of people who have never committed sexual offenses. Therefore, the present meta-analysis seeks to synthesize the evidence from fields such as psychology (Cohen & Galynker, 2012), psychiatry (Carvalho, 2018) neurobiology (Schiffer et al., 2017; Weidacker et al., 2022), or social work (Levenson et al., 2017) to identify factors that distinguish individuals who have never committed sexual offenses against children from those who have.

### Sexual Interest in Children: Prevalence and Characteristics

Bártová et al. (2021), found hebephilic interest in 9.1% (16.8% of men; 1.4% of women), and pedophilic interest in 1.3% (2.3% of men; 0.4% of women) of a representative Czech sample (*N* = 10,044). In the majority of cases, people with a sexual interest in children are also sexually attracted to adults. In Dombert et al.’s study (2,016), only 0.1% reported an attraction to children below the age of 12 that was stronger than their attraction to adults (*n* = 12 out of 8,718). Ephebophilic interest is likely to be common (Seto, 2017), although it may be socially frowned upon (Bennett et al., 2015). Also, as the age of consent may be as low as 14 or 16 in some countries, sex with an adolescent in the ephebophilic range is not always illegal. In some instances, pedophilic interests are considered pathological. To meet the DSM-5 criteria for pedophilic disorder, individuals must be 16 and older, and at least five years older than the child(ren) of interest; present with arousing fantasies, urges, or behaviors towards prepubescent children; and must have acted out on or experienced significant distress or difficulty due to their desires (American Psychiatric Association, 2013). Similarly, the ICD-11 (World Health Organization, 2022) defines pedophilic disorder as persistent and intense sexual interest in prepubertal children, requiring that the individual has either acted on these urges or experiences marked distress, while explicitly excluding age-appropriate sexual behavior among peers. Hebephilic or ephebophilic interests are not considered diagnoses in the DSM-5 or ICD-11.

There is an ongoing debate over the appropriate terminology to use when studying people with a sexual interest in children (Jahnke, Blagden, & Hill, 2022). “Pedophile” is perceived by some as stigmatizing, incorrectly associated with CSA, and too limited, as it only includes sexual attraction to prepubescents (Chamandy, 2020; Martijn et al., 2021). However, alternative terms like “minor-attracted person” or “child-attracted person” have also been criticized for being euphemistic or vague (Jahnke, Blagden, & Hill, 2022). This is because the meaning of “minor” varies across legislations, while the term “child” may both define a person up until the point of puberty and a person that is under the age of consent or under the age of legal majority, depending on the context. In the present review, we will adopt both (a) a broad view of child attraction that includes the spectrum of sexual attraction to children up to ephebophilia, which we will refer to as pedohebephebophilic (PHE), and (b) a narrower view of child attraction that only includes pedophilia and hebephilia, which we refer to as pedohebephilic (PH). Furthermore, following Cohen et al. (2018), we will refer to them as PH/PHE actors and PH/PHE non-actors.

### Sexual Offending: Child Sexual Abuse and Child Sexual Abuse Material

CSA is a contact offense, involving physical engagement in sexual activity with a child. Prevalence of CSA is high on a global scale, with somewhere between 8%–31% of girls, and between 3%–17.6% of boys having experienced CSA (Barth et al., 2013; Pereda et al., 2009; Stoltenborgh et al., 2015). Child sexual abuse material (CSAM) is a non-contact offense which refers to the use of images or videos depicting a child engaged in sexual activity. While it is difficult to ascertain the true prevalence of CSAM use, the National Center for Missing and Exploited Children (2022) received nearly 32 million reports of the possession, production, or distribution of CSAM in 2022. Furthermore, population-based studies in Germany (Dombert et al., 2016) and Czechia (Bártová et al., 2021) both found a prevalence of CSAM use of 2.4%.

Researchers have proposed several frameworks through which to categorize and understand risk factors for committing CSA or CSAM offenses. Finkelhor (1984) developed a four-factor model to conceptualize the motivation to sexually offend against children. The factors represent a sexual interest in children, emotional congruence with children, intimacy deficits/blockage in adult relationships, and overcoming inhibition. Marshall and Barbaree (1990) proposed an integrated model for sexual offending entailing that insecure attachment styles lead to intimacy deficits in adulthood which in turn can lead to loneliness, an aggressive disposition, and ultimately to a tendency to seek intimacy through sexuality and sexual experiences with non-threatening partners. Inspired in part by Finkelhor’s four-factor model (1984), Seto’s Motivation-Facilitation Model (2008, 2017) presents the etiology of sexual offending as stemming from an interaction between traits and states that motivate and facilitate sexual offending. The motivating factors are traits such as paraphilias, originally only pedophilia, but expanded by Seto (2017) to include hebephilia, biastophilia, non-consensual sexual sadism, exhibitionism, and voyeurism, hypersexuality/increased sexual desire, and intense mating effort; while the facilitating factors are divided into traits such as self-regulation problems, hostile masculinity, and states such as negative affect and alcohol use. Seto (2017) further highlights the importance of situational factors such as access to vulnerable victims, the presence of guardians, and time and place.

Although our synthesis integrates findings across the broader spectrum of sexual offending research, the extant literature on sexual offending frequently draws a distinction between factors relevant to its onset and those predictive of reoffending. For instance, theories like Seto’s motivation-facilitation model offer a framework that can conceptually bridge both onset and persistence, identifying shared elements like paraphilic interests and self-regulation deficits (Seto, 2017). However, empirical work often highlights specialized influences; sexual adverse childhood experiences, for example, are consistently linked to initial offending (Widom & Massey, 2015), yet demonstrate a weaker association with sexual recidivism (Astridge et al., 2023). Conversely, factors such as engagement with treatment are more commonly emphasized as predictors of subsequent reoffending (Goodley et al., 2021).

### Potential Distinguishing Factors

We use the term distinguishing factors, rather than risk factors because we expect that most if not all research in this area will be based on cross-sectional or retrospective designs. The factors taken into consideration as distinguishing PHE actors from PHE non-actors are numerous, and categorized, in part, based on Seto’s (2008, 2017) Motivation-Facilitation Model.

#### Motivating Factors

Seto’s (2008, 2017) Motivation-Facilitation Model lists paraphilia, intense mating effort, and high sex drive as factors motivating sexual offenses. In the current meta-analysis, we expand the motivating factors to encompass several other variables related to sexual interest in children such as emotional congruence with children and a preferentiality for children, as we consider them proxies for paraphilia. Some research has found that PHE actors are more likely to have a preferential attraction to children (Bailey et al., 2016) while others have not found any significant differences (Kärgel et al., 2017; Weidacker et al., 2022). Sexual attraction to male relative to female children is a contested factor in distinguishing PHE actors and non-actors, as well (Cohen et al., 2018; Kärgel et al., 2017; Schiffer et al., 2017; Wilpert & Janssen, 2020). Emotional congruence with children, in addition to implicit child-like self-concept and attitude toward children, was found to be associated with greater interest in prepubescent children as well as risk of sexual recidivism (McPhail et al., 2018). Factors such as a hypersexuality/increased sexual desire or sexual preoccupation have been thought to act as motivators for acting (Seto, 2017). Other research found that when comparing individuals who have committed CSA vs. CSAM offenses, the former were more likely to have a detached relationship style and to never have been married, but less likely to have problems with sexual preoccupation and sexual self-regulation (Babchishin et al., 2014; Helmus & Hanson, 2007).

#### Facilitating Factors

Seto (2008, 2017) defines facilitation factors as those which can bypass an individual’s inhibition to act on their motivations. He considered self-regulation problems, hostile masculinity, negative affect, and alcohol use as examples of potential facilitators to sexual offending. In the current meta-analysis, we consider the example factors in addition to other factors which meet the criteria for bypassing inhibition. Impulsivity and mental disorders associated with lower impulse control or antisociality, such as substance abuse disorders and borderline, antisocial, and aggressive personality disorders, are likely to facilitate acting (Babchishin et al., 2014; Cohen et al., 2018; Kärgel et al., 2017; Schiffer et al., 2017; Wilpert & Janssen, 2020). Hostility towards women (Malamuth et al., 1995) has been previously linked to sexual offending in general (Seto et al., 2023) but may not apply to child victims. Cognitive distortions such as the belief that sex with adults is good for children have also been found to be tied to recidivism (Helmus et al., 2013; Stermac & Segal, 1989) among people who have committed sexual offenses against children.

#### Situational Factors

Research suggests that individuals who engage in occupations involving frequent contact with children may be at higher risk of acting on their sexual interests due to increased opportunities for proximity and interaction. This aligns with broader theoretical frameworks emphasizing the interaction of individual predispositions and situational opportunities in offending behavior (Seto, 2017). The presence of biological children has also been examined as a potential distinguishing factor, though its value diminishes when controlling for age (Bailey et al., 2016).

#### Other Factors

Younger PHEs might be more prone to acting due to an increased sexual desire and lower impulse control compared to older PHEs (Barbaree et al., 2003; Harris & Hanson, 2004). Male sex has been established as a distinguishing factor for sexual offending in general (Bailey et al., 2016), which may be a result of an increased sexual desire or cultural factors (Freel, 2003). Neurodevelopmental theories of crime suggest that atypical neurodevelopment may increase vulnerability to certain types of offending behavior (Kärgel et al., 2017). Potential differences in brain morphology or functioning between actors and non-actors will not be included in the meta-analysis because these are not part of a standard clinical assessment. For a recent review of neurobiological characteristics of sexual offenders, see Ramião et al. (2023) and Leverett and Tenbergen (2023). However, research on indirect markers of atypical neurodevelopment like handedness or intelligence will be included. Based on the abused-abuser hypothesis, having experienced CSA can potentially act as a motivator for sex offending during adulthood and especially sex offending against children (Jespersen et al., 2009; Salter et al., 2003; Seto & Lalumière, 2010).

### Objectives

The objectives of the current research are to systematically review and synthesize the literature on the distinguishing factors between PHE actors and non-actors. The results of this meta-analysis can be used to inform research and clinical practice involving PHEs. Specifically, it may help inform differentiated targets in treatment for actors and non-actors.

## Methods

This meta-analysis was conducted in alignment with principles of transparency and openness. The study was preregistered on the Open Science Framework (OSF) to ensure methodological transparency and minimize bias. We reported the total number of excluded cases or observations along with the reasons for these exclusions; listed all dependent variables analyzed, regardless of whether results reached statistical significance; specified whether analyses were prespecified prior to data collection or exploratory in nature; and cited prior publication of any data reported in this manuscript to facilitate future meta-analyses. All data required to reproduce the results presented in this paper, along with the analysis code, have been made publicly available. The preregistration, dataset, and code can be accessed via the following repository: [Distinguishing Pedohebephebophilic Actors and Non-Actors: A Meta-Analysis]. These measures reflect our commitment to reproducibility and the open sharing of scientific knowledge.

### Eligibility Criteria

Publications were analyzed for eligibility based on the following inclusion and exclusion criteria.

#### Inclusion Criteria

To be eligible for inclusion in the current meta-analysis, studies needed to: (1) compare PHE actors and PHE non-actors, (2) determine their participants as PHE either by clinical assessment, penile plethysmography, other indirect methods (e.g., viewing time), or self-report, (3) be of a quantitative nature, and (4) be in a language that can be translated in good quality. There were no criteria for the year of publication, country, or setting and we included grey literature. Regarding criteria (1), we included studies in which the target population is described as nepiophilic, pedophilic, hebephilic, ephebophilic, minor-attracted, sexually interested in children, or any other group that may represent PHEs, as defined in this meta-analysis. The PRISMA flowchart can be found in the Online Supplement.

### Exclusion Criteria

Studies were excluded if (1) they were qualitative, (2) assessed PHE actors or non-actors but did not have the data to make a comparison, (3) participants were not PHE, (4) actor/non-actor or PHE status was not appropriately assessed, (5) the study could not be translated in good quality, (6) they required contacting the authors for additional data and the group sample size was <10, (7) the authors could not be reached, (8) the authors could be reached but the data were no longer available, (9) variables were only related to brain morphology or functioning.

### Information Sources and Search Strategy

Database searches were conducted in December 2023 and December 2024 using ProQuest, PsycNet, PSYNDEX, PubMed, and Web of Science and manual searches were conducted via reference lists of included studies, personal networks, and platforms for sex researchers which continued up until the submission of the first draft. The database search terms utilized were:

(pedophil* OR paedophil* OR nepiophil* OR hebephil* OR pedohebephil* OR ephebophil* OR “minor-attract*” OR “child-attract*”) AND (“non-offen*” OR “sex offen*” OR crim*)

### Study Selection and Data Collection

The literature was screened entirely by two coders, with good agreement for the abstract and title screening (k = .62) and moderate agreement in the full-text screening (k = .47). The extraction and coding were done by the first coder with double coding of 20% of studies from the second coder. Disagreements about inclusions were resolved through discussion. The data extracted and coded included the following study characteristics: type of publication (peer-reviewed journal article, book or book chapter, dissertation or thesis, report, and other), year of publication, setting (clinical, ad hoc community, population-based, forensic, mixed, other), country, language, type of assessment of PHE (self-report, clinical assessment, penile plethysmography, viewing time or other indirect methods, mixed, other), type of effect size (Hedges’ *g* or estimated based on other statistical parameters) in addition to data (means, standard deviations, sample sizes, item sample sizes, and effect sizes) on all of the potential distinguishing factors mentioned, as well as other psychologically meaningful factors that emerged in the screening and extraction process. Because of the breadth of the meta-analysis, the measures of all the distinguishing factors across all the studies reporting them cannot be succinctly reproduced. They are, however, reported in the dataset which is publicly available: [Full Dataset]. We used Hedges’ *g* as an effect size indicator to correct for bias in small or unequal samples. If Hedges’ *g* was not included in the publication, it was determined based on means, standard deviations, and sample sizes. If Hedges’ g could not be calculated, we converted other available effect sizes (Cohen’s *d*, log odds ratio, or *r*) to Hedges’ *g* (Borenstein et al., 2021; Card, 2011).

#### Quality Assessment

We adapted the quality assessment tool from Geraghty and Woodhams (2015) which was based on the Critical Appraisal Skills Programme Tools (2013), the Effective Public Health Practice Project (2008), and the Centre for Review and Disseminations (2009). Changes were made to account for the different populations and outcomes. No publication was excluded based on the results of the quality assessment. The quality assessment tool can be found in the Online Supplement.

### Data Synthesis

The authors take responsibility for the integrity of the data, the accuracy of the data analyses, and have made every effort to avoid inflating statistically significant results.

#### Meta-Analytical Model

Fixed-effects meta-analyses assume that the true effect does not vary from study to study, while random-effects meta-analyses allow for heterogeneity based on the assumptions that effect sizes can differ due to real differences between the studies (Borenstein et al., 2021). As the assumption of a common effect is unrealistic in most practical circumstances, random-effects models have been suggested as the default model for meta-analyses (Borenstein et al., 2010). Following the decision flowchart in Tufanaru et al. (2015), random-effects models were used for five or more studies as fewer studies would not support generalizations beyond the studies included in the meta-analysis. In the latter case, fixed-effects meta-analyses were conducted, the results of which cannot be generalized and must only be applied to the included studies.

Traditionally, effects synthesized with meta-analysis need to be independent, that is, each study can only contribute one effect size. We expected that this assumption would be violated in some cases, as studies may contribute several effect-sizes (e.g., links between one factor and several types of sexual offenses such as CSA, CSAM, and mixed). For both the fixed-effects and random effects meta-analyses, we averaged dependent effect sizes within the study before meta-analyzing them using the *metafor* package in R (Viechtbauer, 2010). We used restricted maximum likelihood (REML) estimation to calculate heterogeneity, as recommended by Viechtbauer (2005).

#### Techniques for Small Study Effects

In meta-analyses, the presence of small study effects can indicate publication bias. To identify such effects, for sample sizes of five or more (Sterne et al., 2000), we visually analyzed funnel plot asymmetry. Additionally, we used an Egger’s test of the intercept (Egger et al., 1997) as a statistical test for funnel plot asymmetry.

#### Subgroup Analysis

We explored via subgroup analyses whether distinguishing factors may relate differently to CSA vs. CSAM. We also conducted exploratory subgroup analyses based on categorization of child-attraction: a broader approach whereby we include minor-attraction even if that means including ephebophilic respondents; and a narrower approach where we include only hebephilic and/or pedophilic samples due to the more atypical nature of these interests compared to ephebophilia.

#### Missing Data

In the event that data were missing from key analyses (e.g., about an effect size of interest) in the included studies, we solicited information from scholars if the research was published after the year 2000 as we expected that it would be unlikely to obtain earlier data. To allow for a relevant comparison, we only contacted authors if comparison groups had more than ten participants. If the requested data could not be obtained or estimated, it was excluded from statistical analyses.

## Results

### Descriptive Information

**Figure 1.**
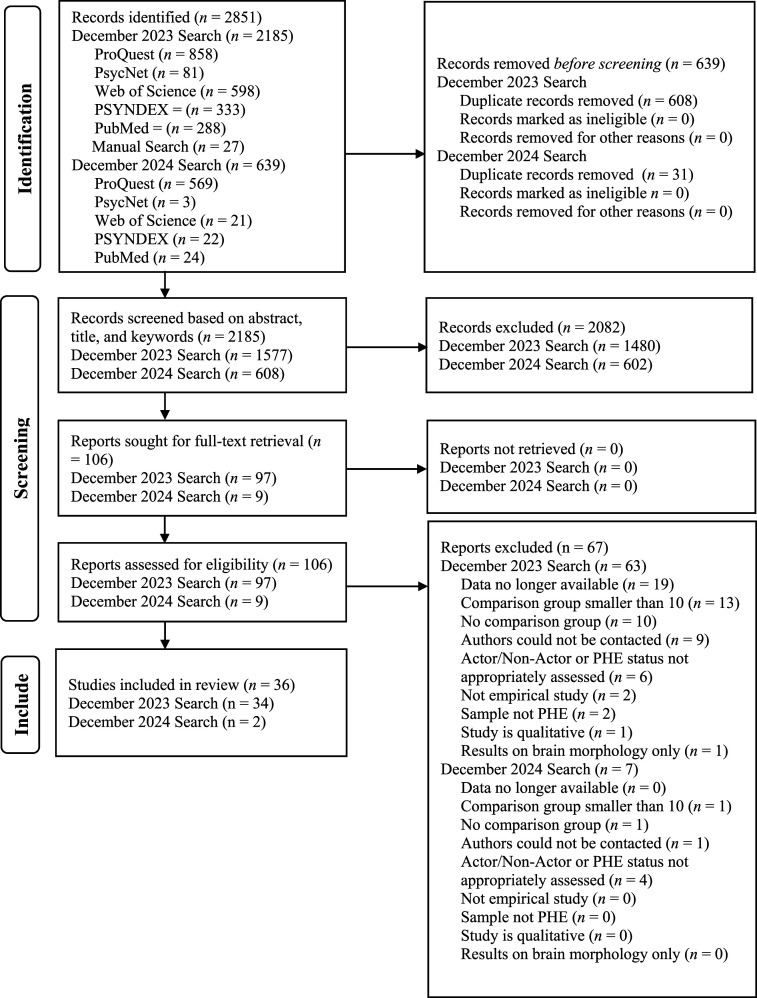
PRISMA Flowchart

Two database searches were conducted, one in December 2023, and another in December 2024. The first database search yielded 2,158 publications (see Figure 1); 608 duplicates were removed and 1480 records were excluded based on abstract, title, and keywords, leaving 97 full-texts assessed for eligibility. The December 2024 database search (see Figure 1) yielded another 608 studies to screen, two of which were included in the final analysis. Most studies included were conducted internationally (33%) in community settings (44%) with male pedohebephilic (58.8%) actors both detected and undetected by law enforcement (35.3%) for CSA or CSAM offenses (64.7%). Several studies stemmed from the same dataset and overlap was clarified with the authors, where possible. Where not possible, the largest sample size was included. Please see Table 1 for study characteristics.

**Table 1.** Study Characteristics

Dataset	Study	Country	Setting	Detection	Attraction	Offense type	Actors *n*	Non-actors *n*	% female	Quality score
1	Adebahr et al. (2021)	Sweden	Clinical	Unspecified	Wide	Mixed	173	49	.04	10
2	Amelung et al. (2024)	Germany	Clinical	Both	Narrow	Mixed	390	32	0	12
3	Babchishin et al. (2017)	Canada	Clinical	Both	Narrow	Mixed	90	29	0	12
4	Bailey et al. (2016)	International	Community	Detected	Wide	Mixed	168	934	0	12
5	Bártová et al. (2021)	Czechia	Population	Unspecified	Narrow	CSAM	369	560	8.2	10
6	Beier, Amelung, et al., 2015	Germany	Clincial	Both	Narrow	Mixed	162	8	0	10
	Beier, Scherner, et al., 2015						59	16		9
7	Cohen et al. (2018)	International	Community	Both	Narrow	Mixed	223	342	4.6	11
8	De Tribolet-Hardy et al. (2024)	Switzerland	Clinical	Both	Narrow	Mixed	36	51	0	10
9	Dombert et al. (2016)	Germany	Population	Both	Narrow	Mixed	157	204	0	15
	Klein et al. (2015)									12
	Koops et al. (2017)									10
	McPhail & Schmidt (2023)									11
	Turner et al. (2016)									10
10	Erkan et al. (2024)	International	Community	Unspecified	Narrow	CSA	33	9	100%	11
11	Geradt et al. (2018)	International	Community	Unspecified	Narrow	Mixed	28	76	0	9
	Jahnke et al. (2015)									13
12	Jahnke & Malon (2018)	International	Community	Detected	Narrow	Mixed	160	23	0	14
13	Jahnke et al. (2024a)	International	Community	Both	Narrow	Mixed	53	230	13.6	11
	Jahnke et al. (2024b)									10
										11
14	Jahnke, Schmidt, et al., 2022	International	Community	Detected	Narrow	Mixed	30	71	0	14
	Jahnke, Schmidt, & Hoyer, 2023									15
15	Jahnke, Schmidt, et al., 2022	International	Community	Detected	Narrow	Mixed	39	239	0	14
	Jahnke, Schmidt, & Hoyer, 2023									15
16	Jahnke et al. (2017)	Germany	Community	Detected	Narrow	Mixed	17	103	0	14
	Jahnke et al. (2019)						17	106		11
17	Konrad et al. (2017)	Germany	Clinical	Both	Narrow	Mixed	383	19	0	12
	Konrad et al. (2018)						239	30		10
18	McPhail & Stephens (2024)	Canada	Community	Detected	Narrow	Mixed	-	-	9.1	9
19	Mitchell & Galupo (2015)	International	Community	Both	Narrow	CSA	31	69	0	11
	Mitchell & Galupo (2018a)						29	71		11
	Mitchell & Galupo (2018b)						22	47		10
20	Schaefer et al. (2010)	Germany	Mixed	Both	Narrow	Mixed	63	97	0	12
21	Schuler et al. (2021)	Germany	Clinical	Both	Narrow	Mixed	652	2,629	-	8
22	Stephens et al. (2023)	International	Community	Detected	Narrow	Mixed	39	172	2.3	13
23	Von Franque et al. (2023)	Germany	Clinical	Both	Narrow	Mixed	148	17	1.81	10
24	Wilpert & Janssen (2020)	Netherlands	Clinical	Both	Narrow	Mixed	166	146	5.4	8

*Note.* The quality assessment (QA) score had a maximum possible score of 16. Please see Supplement 2 for detailed descriptions of each item.

**Table 2.** Distinguishing Factors

Variable	*k*	Model	Hedges’ *g*	Lower CI	Upper CI	Lower PI	Upper PI	*p*	*I* ^*2*^
Motivating Factors									
Any paraphilia	5	Fixed	0.15	0.03	0.27	-	-	.017	73.63%
	5	Random	0.00	−0.32	0.33	−1.08	1.09	.981	74.96%
Emotional congruence with children	2	Fixed	0.14	−0.12	0.39	-	-	.301	0%
	2	Random	0.14	−0.12	0.39	−1.53	1.81	.301	0%
History of falling in love with children	2	Fixed	0.28	0.13	0.43	-	-	<.001	0%
	2	Random	0.28	0.13	0.43	−0.67	1.23	<.001	0%
Hypersexuality/Increased sexual desire	6	Fixed	0	−0.10	0.11	-	-	.986	19.48%
	6	Random	0.03	−0.15	0.20	−0.39	0.44	.759	35.67%
Preferentiality for children	12	Fixed	0.24	0.14	0.33	-	-	<.001	22.32%
	12	Random	0.23	0.12	0.35	0.01	0.46	<.001	17.09%
Preferentiality for male children	5	Fixed	0.12	−0.02	0.26	-	-	.104	65.21%
	5	Random	0.05	−0.24	0.33	−0.87	0.96	.748	63.35%
Facilitating Factors									
Antisocial behavior	5	Fixed	0.25	0.02	0.48	-	-	.035	63.14%
	5	Random	0.26	−0.16	0.68	−1.14	1.65	.228	65.75%
Cognitive distortions	7	Fixed	0.20	0.10	0.31	-	-	<.001	36.47%
	7	Random	0.22	0.08	0.37	−0.1	0.55	.003	28.97%
Empathy	3	Fixed	0.03	−0.27	0.32	-	-	.863	0%
	3	Random	0.03	−0.27	0.32	−1.86	1.92	.863	0%
Hostility	3	Fixed	0.21	−0.08	0.50	-	-	.155	0%
	3	Random	0.21	−0.08	0.50	−1.68	2.1	.155	0%
Impulsivity	2	Fixed	0.13	−0.07	0.33	-	-	.204	0%
	2	Random	0.13	−0.07	0.33	−1.17	1.43	.204	0%
Lack of control of sexual impulses	4	Fixed	0.34	0.20	0.48	-	-	<.001	38.73%
	4	Random	0.43	0.17	0.69	−0.51	1.38	.001	44.07%
Paranoid ideation	3	Fixed	−0.02	−0.31	0.27	-	-	.904	0%
	3	Random	−0.02	−0.31	0.27	−1.91	1.87	.904	0%
Psychoticism	3	Fixed	0.17	−0.12	0.47	-	-	.240	0%
	3	Random	0.17	−0.12	0.47	−1.72	2.07	.240	0%
Substance abuse	3	Fixed	0.23	−0.26	0.72	-	-	.364	0%
	3	Random	0.23	−0.26	0.72	−2.96	3.42	.364	0%
Situational Factors									
Contact with children	4	Fixed	0.17	0.04	0.30	-	-	.010	72.14%
	4	Random	0.06	−0.29	0.41	−1.48	1.6	.750	79.01%
Own children	9	Fixed	0.26	0.15	0.37	-	-	<.001	0%
	9	Random	0.27	0.15	0.39	0.09	0.45	<.001	4.68%
Other Factors									
ACEs – Non-sexual	4	Fixed	0.38	0.23	0.53	-	-	<.001	0%
	4	Random	0.38	0.23	0.53	0.05	0.71	<.001	0%
ACEs – Sexual	9	Fixed	0.35	0.26	0.45	-	-	<.001	58.3%
	9	Random	0.38	0.22	0.54	−0.08	0.84	<.001	56.68
Affective disorders	7	Fixed	0.05	−0.10	0.19	-	-	.543	0%
	7	Random	0.05	−0.10	0.19	−0.15	0.24	.543	0%
Age	18	Fixed	0.43	0.36	0.49	-	-	<.001	76.46%
	18	Random	0.50	0.36	0.65	−0.05	1.06	<.001	74.9%
Age of onset of sexual interest	3	Fixed	−0.01	−0.14	0.12	-	-	.899	0%
	3	Random	−0.01	−0.14	0.12	−0.86	0.84	.898	0.03%
Agreeableness	2	Fixed	−0.24	−0.72	0.25	-	-	.338	0%
	2	Random	−0.24	−0.72	0.25	−3.36	2.89	.338	0%
Anxiety	6	Fixed	0.02	−0.13	0.17	-	-	.775	0%
	6	Random	0.02	−0.13	0.17	−0.19	0.24	.775	0%
Any personality disorder	2	Fixed	−0.20	−0.71	0.31	-	-	.439	0%
	2	Random	−0.20	−0.71	0.31	−3.48	3.08	.439	0%
Attention deficit	2	Fixed	0.05	−0.64	0.73	-	-	.895	0%
	2	Random	0.05	−0.64	0.73	−4.38	4.47	.895	0%
Conscientiousness	2	Fixed	−0.05	−0.53	0.43	-	-	.831	0%
	2	Random	−0.05	−0.53	0.43	−3.18	3.07	.831	0%
Distress – General	6	Fixed	0.13	−0.04	0.30	-	-	.124	0%
	6	Random	0.13	−0.04	0.30	−0.11	0.37	.124	0%
Distress - sexual interest	3	Fixed	0.21	0.11	0.30	-	-	<.001	13.22%
	3	Random	0.21	0.11	0.30	−0.42	0.84	<.001	0.01%
Education	13	Fixed	0.02	−0.07	0.12	-	-	.674	0%
	13	Random	0.02	−0.07	0.12	−0.09	0.13	.674	0%
Extraversion	2	Fixed	−0.12	−0.60	0.36	-	-	.619	0%
	2	Random	−0.12	−0.60	0.36	−3.25	3	.619	0%
Head injuries after age 13	2	Fixed	0.34	0.070	0.61	-	-	.015	0%
	2	Random	0.34	0.070	0.61	−1.41	2.09	.015	0%
Head injuries before age 13	2	Fixed	0.02	−0.25	0.28	-	-	.900	0%
	2	Random	0.02	−0.25	0.28	−1.71	1.75	.900	0%
Height	2	Fixed	−0.35	−0.62	−0.07	-	-	.013	24.92%
	2	Random	−0.34	−0.66	−0.02	−2.88	2.2	.037	24.92%
Intelligence	2	Fixed	−0.86	−1.18	−0.53	-	-	<.001	0%
	2	Random	−0.86	−1.18	−0.53	−2.95	1.24	<.001	0%
Intimate partner	8	Fixed	0.11	−0.04	0.25	-	-	.156	58.48%
	8	Random	0.07	−0.16	0.30	−0.58	0.72	.565	54.82%
Living alone	2	Fixed	−0.12	−0.42	0.17	-	-	.414	0%
	2	Random	−0.12	−0.42	0.17	−2.06	1.81	.414	0%
Loneliness	3	Fixed	0.16	−0.1	0.41	-	-	.232	60.84%
	3	Random	0.13	−0.32	0.58	−4.74	5	.572	62.12%
Male sex	8	Fixed	0.43	0.23	0.64	-	-	<.001	50.45%
	8	Random	0.53	0.17	0.90	−0.46	1.52	.004	55.52%
Neuroticism	2	Fixed	−0.07	−0.55	0.41	-	-	.783	0%
	2	Random	−0.07	−0.55	0.41	−3.19	3.06	.783	0%
Non-heterosexuality	7	Fixed	0.21	0.08	0.33	-	-	.002	0%
	7	Random	0.20	0.06	0.34	−0.02	0.42	.005	5.61%
Non-right handedness	3	Fixed	0.19	0.03	0.35	-	-	.018	0%
	3	Random	0.19	0.03	0.35	−0.83	1.21	.018	0%
Obsessive-compulsive	3	Fixed	0.08	−0.21	0.37	-	-	.605	0%
	3	Random	0.08	−0.21	0.37	−1.81	1.97	.605	0%
Openness	2	Fixed	−0.31	−0.79	0.17	-	-	.208	37.24%
	2	Random	−0.45	−1.30	0.39	−8.15	7.24	.293	37.24%
Other mental health diagnosis	4	Fixed	0.05	−0.20	0.30	-	-	.706	59.67%
	4	Random	0.04	−0.38	0.46	−1.6	1.69	.843	57.65%
Phobia	3	Fixed	0.13	−0.17	0.42	-	-	.401	0%
	3	Random	0.13	−0.17	0.42	−1.77	2.02	.401	0%
Self-esteem	2	Fixed	0.10	−0.06	0.26	-	-	.205	0%
	2	Random	0.10	−0.06	0.26	−0.93	1.13	.205	0%
Social desirability	4	Fixed	0.15	−0.08	0.37	-	-	.196	0%
	4	Random	0.15	−0.08	0.37	−0.35	0.64	.196	0%
Somatization	4	Fixed	0.04	−0.17	0.25	-	-	.724	52.79%
	4	Random	0.10	−0.23	0.43	−1.15	1.36	.538	52.34%
Stigma	4	Fixed	0.61	0.53	0.68	-	-	<.001	97.15%
	4	Random	0.21	−0.24	0.66	−1.91	2.33	.362	95.16%
Stigma (without outlier)	3	Fixed	−0.02	−0.16	0.12	-	-	.748	0%
	3	Random	−0.02	−0.16	0.12	−0.93	0.89	.748	0%
Suicidality	4	Fixed	0.00	−0.22	0.21	-	-	.987	0%
	4	Random	0.00	−0.22	0.21	−0.47	0.47	.987	0%
Therapy – Attendance	8	Fixed	0.28	0.14	0.43	-	-	<.001	73.81%
	8	Random	0.43	0.04	0.82	−0.79	1.66	.029	80.63%
Therapy – Attendance (without outlier)	5	Fixed	0.11	−0.06	0.27	-	-	.195	0%
	5	Random	0.11	−0.06	0.27	−0.16	0.38	.195	0%
Therapy – Interest	4	Fixed	0.43	0.26	0.59	-	-	<.001	91.39%
	4	Random	0.45	−0.28	1.18	−3.02	3.92	.224	94.11%
Therapy – Interest (without outlier)	2	Fixed	−0.02	−0.27	0.23	-	-	.878	74.4%
	2	Random	−0.09	−0.61	0.43	−5.43	5.25	.732	74.4%
Unemployment	6	Fixed	0.03	−0.16	0.21	-	-	.773	36.71%
	6	Random	0.04	−0.19	0.27	−0.51	0.6	.713	31.73%

*Note.* Positive effect sizes indicate that a factor is associated with acting, while negative effect sizes indicate that a factor is associated with non-acting. Both fixed-effects and random-effects models are presented to allow for comparisons. While random-effects models were the preferred approach for meta-analyses including five or more studies, fixed-effects models may be appropriate when fewer than five studies are available.

### Quality Assessment

The studies had a mean quality score of 11.13 (*SD* = 1.82) out of 16. Very few studies recruited samples representative of the target population (17%), took missing data into consideration (29%), or took into account potential confounders (37%). The quality assessment items, and inter-rater reliability can be found in the Online Supplement.

### Meta-Analytic Findings (see Table 2)

#### Motivating Factors

A preferentiality for children was found to be a significant distinguishing factor based on 11 studies as actors were more likely to exhibit a stronger preference for children. A history of falling in love with children was also more common among actors. A preference for male children was not found to be a significant predictor of acting based on a random-effects model with five studies, nor was an emotional congruence with children. Any paraphilia other than PHE did not emerge as a distinguishing factor, nor did hypersexuality/increased sexual desire. The heterogeneity in this group varies, with high levels observed for any paraphilia (I^2^ = 74.96%), having an intimate partner (I^2^ = 66.96%), and preferentiality for male children (I^2^ = 63.35%). Moderate heterogeneity is seen in hypersexuality (I^2^ = 41.60%) and low in preferentiality for children (I^2^ = 19.61%).

#### Facilitating Factors

Actors were found to exhibit lower levels of control over sexual impulses compared to non-actors and to have significantly more cognitive distortions. Other potential facilitating factors examined, including antisocial behavior, empathy, hostility, impulsivity, paranoid ideation, psychoticism, and substance abuse were not found to significantly differ between actors and non-actors. Moderate heterogeneity is observed for cognitive distortions (I^2^ = 28.97%). The remainder of the facilitating factors were insufficiently powered to discuss heterogeneity.

#### Situational Factors

Contact with children and having own children both emerged as significant factors, highlighting the role of situational facilitation in the decision to act.

#### Other Factors

Actors were observed to be older on average compared to non-actors, male, non-heterosexual (in terms of attraction to adults), and to have sustained head injuries after the age of 13 (but interestingly, not before age 13). The age of onset of PHE attractions were not distinguishing characteristics. While handedness did not differ significantly between the groups, height and intelligence emerged as distinguishing characteristics, with actors tending to be shorter and less intelligent than non-actors.

Socioeconomic circumstances such as living alone, education and employment were not significant, nor were mental health factors (affective disorders, anxiety, general distress, phobias, self-esteem, social desirability, somatization, suicidality), other mental health diagnoses, attention deficit, any personality disorders, or personality factors (agreeableness, conscientiousness, extraversion, neuroticism, openness, obsessive-compulsive traits). Actors were more likely to experience distress related to their sexual interest in children, be interested in, and have engaged in, therapy, and to experience greater stigma, however, the effect of stigma did not hold when controlling for outliers.

Both sexual adverse childhood experiences and non-sexual adverse childhood experiences emerged as significant factors in distinguishing PHE actors from non-actors, with non-actors reporting significantly fewer instances of both sexual and non-sexual trauma. High heterogeneity is evident for age (I^2^ = 77.14%) and therapy attendance (I^2^ = 80.63%). Moderate heterogeneity is observed for non-heterosexuality (I^2^ = 5.61%) and male sex (I^2^ = 8.04%), indicating slightly variable effects. Low or no heterogeneity is seen for factors like education (I^2^ = 0.1%) and anxiety (I^2^ = 0%), reflecting consistent findings across studies. Neither loneliness nor having an intimate partner were significant in distinguishing actors from non-actors.

#### Subgroup Analysis

Subgroup analyses were performed on studies with a narrow definition of sexual interest in children, excluding ephebophilia, studies looking at CSAM actors vs. non-actors, and studies looking at CSA actors vs. non-actors (see Online Supplement). The CSAM and CSA analyses significantly reduced the number of viable studies per analysis, limiting most factors to fixed-effects meta-analyses, while only two studies featured ephebophilia (Adebahr et al., 2021; Bailey et al., 2016), ultimately rendering the pedohebephilia subgroup analysis a brief sensitivity analysis. Preferentiality for male children became significant in the CSAM only and the CSA only subgroup, any paraphilia became significant in the CSAM only subgroup and the PH only subgroup, and unemployment became significant in the CSA only subgroup.

#### Small Study Effects

Education resulted in a significant Egger’s test (*p* = .03); however, visual inspection of the funnel plot did not indicate an effect. Neither visual inspection of funnel plots nor Egger’s test (see Online Supplement) indicated small study effects for any other variables.

## Discussion

This literature review identified 22 studies that allowed for investigating differences between actors and nonfactors on a large range of factors. For a total of 54 potential distinguishing factors, it was possible to calculate average differences using meta-analysis. Out of these, 16 meta-analyses were based on sufficient primary studies to allow for random-effects meta-analyses. Amongst these, eight found significant average effects associated with acting, which were largest for male sex, prior therapy, older age, and adverse childhood sexual experiences (all *g*s > .38). Small significant effects were discovered for having own children, being preferentially attracted to children, cognitive distortions, and non-heterosexual orientation (all *g*s > .20). It is important to emphasize that the results related to non-heterosexuality do not show that non-heterosexuality is a risk factor for any harmful behaviors or that individuals identifying as gay, bisexual, or otherwise pose any inherent risk to children or others. While non-heterosexual orientations might be a marker of risk among people who are sexually attracted to children, more research is needed to rule out alternative explanations (such as confounds introduced by combining forums with different cultures and different focuses on boys, girls, or children of both sexes). For the 34 remaining factors, effect sizes were based on fewer than 5 studies and fixed-effects meta-analysis. While these results need to be interpreted with caution, results were significant for intelligence, stigma, interest in therapy, nonsexual adverse childhood experiences, height, lack of control of sexual impulses, head injuries after age 13, a history of falling in love with children, distress about sexual interests, non-righthandedness, and contact with children. These factors represent promising avenues for future research. Average effect sizes for personality factors, mental disorders (e.g., affective or anxiety disorders), impulsivity or distress were very small (below *g* = .20) and non-significant. These findings have implications for theoretical understanding and practical approaches to treatment.

### Theories of Child Sexual Offending

Prominent theories of child sexual offending consider sexual attraction to children only as one of several factors that can increase or decrease the risk of child sexual offending. Many theories of child sexual offending posit that the presence or absence of additional factors like the opportunity to offend or intimacy and self-regulation problems would determine the sexual offending risk of people who are sexually attracted to children (Finkelhor, 1984; Marshall & Barbaree, 1990; Seto, 2008, 2017). An alternative perspective suggests that factors may have differential effects for people with or without a sexual interest in children, implying the presence of moderation effects. While the findings of the present meta-analysis corroborate the importance of many of these factors among people with a sexual interest in children (lack of control of sexual impulses, access to children), other factors could not be confirmed (emotional congruence with children, intimacy deficits, attachment style, loneliness, hostility).

While the meta-analysis identified common factors associated with higher perpetration rates across various criminal behaviors—including cognitive distortions (Syasyila et al., 2024), sexual and non-sexual adverse childhood experiences (Fox et al., 2015; Malvaso et al., 2022; Vachon et al., 2015), low intelligence (Jovanovic et al., 2012), and male sex (Gartner, 2011)—other factors demonstrated notable specificity. Specifically, factors related to pedohebephebophilic interest, such as a history of falling in love with children and a preferentiality for children, were highly indicative of sexual offending. Interestingly, older age, while generally linked to decreased general criminality (McCall et al., 2013), suggested persistent or new-onset risk for sexual offenses. Subgroup analyses provided further insights into this specificity: preferentiality for male children became significant in studies focusing solely on CSAM actors and CSA actors, while any paraphilia was significant in CSAM-only and pedohebephilia-only subgroups, although only based on fixed-effects models.

### Motivating Factors

Substantiating the importance of sexual attractions as a motivating factor for child sexual offending, the present meta-analysis found that preferential attraction to children was associated with a higher risk to commit child sexual offenses. However, two previously identified studies (Kärgel et al., 2017; Weidacker et al., 2022) could not be included in the meta-analysis due to their classification of CSAM users/hands-off offenders as non-actors. These studies did not find evidence for preferential attraction to children. For emotional congruence with children as another factor posited to motivate sexual attraction to children, the results did not corroborate a significant association with acting, which is inconsistent with Finkelhor’s four factor model (1984). A history of falling in love with children, on the other hand, was significantly related to acting. Feelings of romantic love are conceptually linked to an emotional congruence with children, in the sense that both reflect emotional aspects of contact with children (Seto, 2012). Compared to emotional congruence with children, the experience of love may be a factor that is more directly/proximally related to sexual offending. In accordance with the motivation-facilitation model, it is plausible to assume that stronger sexual and romantic interests represent the motivational component of child sexual offending. Preferentiality for male children was not significantly related to child sexual offending in the meta-analysis. While gender preferences with regards to children are likely to affect victim choice, they may not be an important determinant for acting per se. This is in opposition to evidence from forensic samples showing that the choice of male victims is associated with a higher recidivism rate (Hanson & Bussière, 1998). This contradiction may be explained by a statistical artifact in forensic samples, whereby male child victims are more common among people with a primary sexual attraction to children than among people with a primary sexual attraction to adults (Seto et al., 2017).

Surprisingly, neither additional paraphilias nor hypersexuality/increased sexual desire were corroborated as motivating distinguishing factors. While paraphilias were found to be predictors of sexual recidivism in previous meta-analysis (Hanson & Morton-Bourgon, 2005), it is possible that these factors only add very little to the already increased risk of acting among people with a sexual interest in children. Of note, hypersexuality is linked with paraphilic interests, including attraction to children (Jahnke et al., Manuscript in Preparation). Hence, the link between hypersexuality and sexual offending may be masked among people with a sexual interest in children, as it might be that the connection between the two is mediated by the attraction itself. Intimacy deficits or blockage of adult relationships have a long-standing history in theorizing about child sexual offending (Finkelhor, 1984). However, the present review did not find higher rates of loneliness or of not having an intimate partner in actors vs. non-actors. It is possible that intimacy deficits are more pertinent factors for people who normally desire and seek adult partners and who may commit sexual offenses against children when their preferred sexual partners appear unattainable. There is a lack of studies assessing the role of intimacy deficits or blockage, as proposed by Finkelhor (1984).

### Facilitating Factors

The significant difference in cognitive distortions between pedohebephebophilic actors and non-actors confirms its long-suspected role in facilitating child sexual offending (Helmus et al., 2013; Stermac & Segal, 1989). Cognitive distortions may develop as a means to alleviate the cognitive dissonance between the desire to have one’s sexual needs fulfilled and societal norms that engaging with children sexually is morally wrong (Eberhaut et al., 2023). One such distortion is the belief that sexual interactions between children and adults are not immoral or harmful (Steel et al., 2020). Actors may frame such actions as expressions of affection or as beneficial to the child, distorting societal norms and ethical standards to align with their desires. Another prevalent distortion involves the belief that children are capable of consenting to sexual activities (Eberhaut et al., 2023). Actors may convince themselves that a child’s compliance or lack of resistance equates to consent, ignoring the inherent power imbalance and the child’s limited understanding of the situation. Similarly, actors might rationalize their actions by perceiving the child as an active participant or even the initiator of the sexual interaction (Abel et al., 1989). Although cognitive distortions can, to some extent, be conceptualized as features of pedophilic attraction (Eberhaut et al., 2023), the present findings suggest that they differentiate between actors and non-actors even among groups of people with sexual attraction to children. The average effect size determined in the present meta-analysis was small, but comparable to average effect sizes from recidivism studies among people who have sexually offended against children (Helmus et al., 2013).

Self-regulation problems, or lack of control of sexual impulses, as described in this meta-analysis, were significantly associated with acting, supporting previous findings on sex offenders’ recidivism (Hanson & Morton-Bourgon, 2005). These findings are consistent with research suggesting that individuals with poor sexual self-regulation struggle to resist immediate gratification (Babchishin et al., 2014), even when such actions have negative long-term consequences while individuals with higher self-regulation capacities are better able to employ strategies to avoid risky situations (Helmus & Hanson, 2007). However, given the cross-sectional nature of the primary studies, it is possible that the perception of lacking sexual self-regulation is informed by previous experiences of losing sexual self-control among actors. It is also of note that, while similar, lack of control of sexual impulses and impulsivity seem to present as distinct characteristics, with the former determined to be a significant factor and the latter not.

The non-significance of empathy as a distinguishing factor is particularly noteworthy, as it does not align with the well-established role of reduced empathy in predicting criminal or harmful behavior (Mann & Barnett, 2013). While more research is needed, it may be that actions are more strongly influenced by cognitive distortions that rationalize or minimize their actions, rather than an inherent inability to empathize. Hostility, paranoid ideation, psychoticism, and substance abuse were also not found to be relevant, though the generalizability of this finding is limited due to the small sample sizes. Overall, the statistically significant facilitating factors appear to enable one another, with cognitive distortions acting to justify the gratification of sexual impulses, as opposed to mental health and personality factors impairing one’s ability to abstain.

### Situational Factors

Opportunity provides the situational context necessary to facilitate acting. One significant source of opportunity is having one’s own children (Bailey et al., 2016). Parents or guardians inherently have regular, unsupervised access to their children and potentially their children’s peers, which can create an environment of trust and authority that is easily exploited. The proximity can lower external barriers to acting, as the actor does not need to seek out or groom a child outside their immediate household. It is unclear whether actors have offended against their biological children, however, and this result could be an artifact of age, as older individuals are more likely to have children and to have offended. Another common source of opportunity is contact with other children (Bailey et al., 2016), such as through employment, volunteer activities, or social and familial relationships. Actors may deliberately seek positions that bring them into close and frequent contact with children, such as teaching, coaching, or caregiving roles. These settings allow actors to build trust with potential victims and their families, providing opportunities for grooming and abuse. In both familial and extrafamilial contexts, access to children may lower the practical barriers to acting and increase the likelihood that an individual might escalate from attraction to action.

### Other Factors

Older individuals may have had more opportunities to act on their interests (Seto, 2017), while the predominance of males among actors reflects broader patterns of sexual offending across genders, potentially influenced by differences in sexual drives or social norms (Freel, 2003). Non-heterosexuality was more common among actors, while age of onset did not differ. Low intelligence and shorter stature have been associated with acting, potentially reflecting neurodevelopmental vulnerabilities or early life adversity that might impair social and interpersonal functioning. Non-right-handedness and a history of head injuries, particularly those sustained after age 13, suggest that neurological anomalies or trauma may play a role in disrupting decision-making processes, increasing the likelihood of acting (Fazio et al., 2014; Jordan et al., 2023). The generalizability of this result is questionable, however, as both factors are based on only two studies. Education level and unemployment did not significantly differentiate actors from non-actors. This aligns with research suggesting that while socioeconomic factors such as education and employment are associated with general offending behavior (Farrington et al., 2017), their influence may be context-dependent and less predictive in specific offending subtypes.

Actors were significantly more likely to have engaged in therapy. However, caution is needed when interpreting this finding, as the variables assessing therapeutic history did not discern whether therapy was sought or mandated in response to sexual offending. Even in the absence of mandated therapy, it is conceivable that individuals with a higher self-perceived offending risk or a previous history of (undetected) acting are more likely to self-refer to mental health treatment than individuals with a lower risk. This aligns with the fact that interest in treatment was higher among actors than among non-actors. Potential differences in the age of people with and without prior therapy experiences were not accounted for. Furthermore, it is unclear whether the sexual interest in children was addressed in treatment, as some clients with a sexual interest in children do not disclose this in treatment (Jahnke et al., 2024a). In addition, few therapists are perceived to have specialized knowledge about sexual attraction to children outside of forensic services, and some may have used harmful or unnecessary interventions (Chronos et al., 2024).

Stigma was notably higher among actors. However, this result was dependent on one highly influential outlier (McPhail & Stephens, 2024), and the results were non-significant after the outlier was removed. Stigma has also been theorized to indirectly affect offending risk (Jahnke, 2018). Notably, there is a lack of evidence that proposed mediating factors such as mental health problems, willingness to seek treatment, or poor social functioning, are associated with a higher risk of sexual offending, including in the current meta-analysis. More sophisticated studies, ideally differentiating between different facets of stigma (Jahnke et al., 2024b) and building on longitudinal designs, are needed to assess the links between stigma, mental health problems, and treatment seeking while controlling for potential confounds like offending risk.

Both sexual adverse childhood experiences and non-sexual adverse childhood experiences emerged as significant factors in distinguishing PHE actors from non-actors, aligning with the broader literature on the impact of early adverse experiences (Jespersen et al., 2009; Seto & Lalumière, 2010), as well as previous meta-analysis on the factors associated with recidivism among sex offenders (Hanson & Morton-Bourgon, 2005). Non-actors reported significantly fewer instances of both sexual and non-sexual adverse childhood experiences. This finding supports the potential protective role of a stable and supportive childhood environment (Babchishin et al., 2014). Some scholars have proposed that sexual adverse childhood experiences contribute to the development of sexual attraction to children and cognitive distortions via conditioning and social learning (Marshall & Marshall, 2000; Schippers et al., 2024). More research is needed to understand the role of potential moderating (e.g., poor attachment and low self-esteem, genetic predisposition) and mediating factors (e.g., believing that CSA is normal, Marshall & Marshall, 2000).

No significant differences emerged among any of the Big Five personality factors, affective disorders, anxiety, general distress, phobia, obsessive-compulsive traits, self-esteem, social desirability, or somatization, despite previous associations between personality traits and maladaptive behaviors (Babchishin et al., 2014; Kärgel et al., 2017). Distress related to sexual interest in children was, however, significantly more pronounced among actors. It is conceivable that their distress was influenced by the repercussions of being detected, and in some instances, convicted of sexual offenses, which would alter the direction of the assumed causal relationship.

### Strengths, Limitations, and Future Directions

The systematic review sought to integrate findings across all manner of participant characteristics, study design, settings, and including grey and unpublished literature. In order to ensure as thorough an overview as possible, this included soliciting effect sizes for comparisons between actors and non-actors that were not part of the published manuscripts. Even though small-study bias could not be tested for most of the meta-analysis due to the small number of studies, this procedure helped to mitigate potential publication biases. The meta-analysis followed a preregistered plan to ensure transparency and minimize the risk of bias.

A key limitation is the small number of studies included for several variables, which precluded conducting moderator analyses for many factors and, consequently, hindered our ability to explore potential sources of heterogeneity. This limitation underscores the challenge of identifying explanatory variables that might account for inconsistencies across studies. Additionally, the majority of studies included were cross-sectional in design, which poses a significant constraint on establishing temporal order. As such, causal relationships remain unclear—for example, whether feelings of stigmatization lead to offending behavior or, conversely, whether such feelings are a consequence of offending. Additionally, due to the cross-sectional nature of the included studies, the current meta-analysis could not explicitly separate factors related to onset and recidivism. This uncertainty complicates the interpretation of findings and highlights the need for longitudinal research.

The validity of the results is also limited by concerns about the quality of primary studies. For instance, most studies did not recruit samples representative of the target population, nor did they take any potential confounders into account (see Online Supplement). Another notable limitation is the variability in study methodologies and definitions across the included research. For example, the scales used to assess factors such as cognitive distortions, stigma, or self-control varied from study to study. Many of the scales were ad hoc without established psychometric properties, raising concerns about their reliability and validity (Elson et al., 2023). The majority were conducted in in Western contexts, and even the international online surveys were rarely available in languages other than English. The majority of the non-acting populations were recruited as online ad-hoc samples where we can expect self-selection of individuals who would have the inclination to participate in research on the topic. Furthermore, although different samples were treated as independent in our meta-analysis, there may be an unknown level of overlap between samples of PHE recruited online and via clinical programs such as the German Prevention Network “Kein Täter Werden” (Don’t Offend) whereby participants partake in several studies. PHE recruited online are likely to be younger, more intelligent, and more educated than the general population (Jahnke, Schmidt, et al., 2022). Hence, it is unclear whether these findings would generalize to PHE individuals in the general population.

Another limitation was the number of studies we could not include in the meta-analysis due to a misalignment of classifications and subsequent inability to either contact or obtain additional data from authors. For instance, data from the NeMUP studies could not be included as CSAM users were classified as non-actors in all associated publications. Hence, the meta-analysis is missing crucial insights from the seminal NeMUP studies which were among the first large-scale attempts to differentiate actors from non-actors (Gerwinn et al., 2018), even though a re-classification of participants might have been possible through secondary data analysis.

### Implications for Clinical Practice

The findings of this meta-analysis provide several implications for clinical practice. The present meta-analysis identified factors that may help distinguish between PHE with higher and lower levels of offending risk. This is likely to affect decision making with regards to how to weigh prevention and well-being focused goals in treatment, whereby treatment for higher-risk individuals should put more weight on addressing factors associated with offending behavior (Bonta & Andrews, 2007). Conversely, for those identified as low risk, a well-being focused approach can prevent unnecessary interventions that are likely to hurt the therapeutic alliance. Similarly, a recent Delphi study by Stephens et al. (2024) provides a consensus on practice guidelines for the assessment and treatment of individuals with sexual interest in children in non-mandated settings. These guidelines emphasize the importance of considering co-occurring mental health conditions while also highlighting disagreements over the use of forensic practices.

Psychologically meaningful risk factors for child sexual offending are dynamic and changeable risk factors that are a plausible cause of sexual offending and that predict recidivism (Mann et al., 2010). Based on this conceptualization, cognitive distortions, a lack of control over sexual impulses, and access to children can be considered promising candidates for psychologically meaningful risk factors. Clinicians can employ cognitive restructuring techniques to address distortions (Friestad, 2012; Moster et al., 2008), and cognitive behavioral therapy or pharmacological treatment, among others, to increase control over sexual impulses (Derbyshire & Grant, 2015). For clients at high risk of sexual offending and access to children, clinicians need to work on strategies to reduce the immediate risk to children. If offending appears likely or inevitable, clinicians need to consider whether they have a legal right or obligation to report their client to the police and/or child protection services (Stephens et al., 2021). However, clients should not be reported only because they are sexually attracted to children (Stephens et al., 2024).

A preferential sexual attraction to children and a history of falling in love with children have also emerged as factors associated with sexual offending. While there is no good evidence that preferentiality can be changed, clients may benefit from techniques that increase behavioral approaches that decrease sexual arousal to children (McPhail & Olver, 2020) or from arousal-reducing medication (Landgren et al., 2022). General mental health problems were not found to be significant factors differentiating between acting and non-acting individuals. Efforts to destigmatize voluntary therapy and expand access to confidential, non-judgmental therapeutic services may encourage individuals to seek help, as has been suggested by previous literature (Chronos et al., 2024).

## Supplemental Material

Supplemental Material - Distinguishing Pedohebephebophilic Actors and Non-Actors: A Meta-Analysis Supplemental Material for Distinguishing Pedohebephebophilic Actors and Non-Actors: A Meta-Analysis by Agatha Chronos and Sara Jahnke in Sexual Abuse
