# Supplementary material for: Distinguishing Pedohebephebophilic Actors and Non-Actors: A Meta-Analysis
Source: Sex Abuse. 2025 Nov 10;38(2):127–62. doi: 10.1177/10790632251389171 (PMC12804425; doi:10.1177/10790632251389171)
Supplement: Supplemental Material - Distinguishing Pedohebephebophilic Actors and Non-Actors: A Meta-Analysis [file sj-pdf-1-sax-10.1177_10790632251389171.pdf]

Online Supplement

Chronos, A. & Jahnke, S. Distinguishing Pedohebephebophilic Actors and Non-Actors: A Meta-Analysis.

Quality Assessment Tool

**Assessment of quality (Only score for relevant items)**	**Guidance for scoring each sub-section**	**Overall rating of quality**
Q1. Were the study objectives clear?	Yes = The objectives were clear.<br>Partially = The objectives were somewhat clear.<br>No or N/A = The objectives were not clear and/or the purpose of the study was something other than to compare PHE actors/non-actors.	Yes = 2<br>Partially = 1<br>No or N/A = 0
Q2. Were the participants recruited in an acceptable way?	Yes = Participants were appropriately selected, recruitment process described, and ethical principles adhered to.<br>Partially = Recruitment process is described but important details are missing.<br>No = No recruitment process described.	Yes = 2<br>Partially = 1<br>No = 0
Q3. Are the individuals selected to participate in the study likely to be representative of the target population?	Yes = Participants from population-based sample.<br>Partially = Systematically selected from a source list (i.e., support organization, clinic, prison, mental health facility) or self-referred from multiple sources.<br>No = Self-referred from only one source or participant characteristics are not appropriately described.	Yes = 2<br>Partially = 1<br>No = 0
Q4. Were all relevant variables clearly defined (offense status, distinguishing factors, child attraction)?	Yes = Clear definition of all or most variables (e.g., what is child-attraction? What constitutes offending, contact offenses, CSAM?)<br>Partially = Clear definition of some variables.<br>No = No definition of any variables or unclear definition.	Yes = 2<br>Partially = 1<br>No = 0
Q5. Were all relevant variables appropriately measured (offense status, distinguishing factors, child attraction)?	Yes = All or most of the methods of data collection are valid and reliable.<br>Partially = Some of the methods of data collection are valid and/or reliable.<br>No = None of the methods of data collection were valid and reliable, or validity and reliability were not described.	Yes = 2<br>Partially = 1<br>No = 0
Q6. Was missing data dealt with appropriately?	Yes = Missing data was reported and considered, was negligible, or there was no missing data, and the study reported this.<br>Partially = Missing data was reported but not taken into consideration.<br>No = Missing data was not reported at all.	Yes = 2<br>Partially = 1<br>No = 0
Q7. Were the appropriate statistical tests used (e.g., t-tests, ANOVA, logistic analysis)?	Yes = All statistical tests used were appropriate for the research design and question.<br>Partially = Some statistical tests used were appropriate for the research design and question.<br>No = Statistical tests used were not appropriate for the research design and question.	Yes = 2<br>Partially = 1<br>No = 0
Q8. Were potential confounders taken into account?	Yes = Any or most of potential confounders were taken into consideration.<br>Partially = Some efforts made to control for confounders.<br>No = No effort made to control for potential confounders.	Yes = 2<br>Partially = 1<br>No = 0
**Quality Score: /16**		

Quality Assessment Results

	Q1	Q2	Q3	Q4	Q5	Q6	Q7	Q8	Total
Adebahr et al., 2021	1	2	1	2	1	1	2	0	10
Amelung et al., 2024	0	2	1	2	2	2	2	1	12
Babchishin et al., 2017	0	2	1	2	2	1	2	2	12
Bailey et al., 2016	2	2	1	2	0	1	2	2	12
Bártová et al., 2021	0	2	2	2	0	2	2	0	10
Beier et al., 2015a	0	2	1	2	2	1	2	0	10
Beier et al., 2015b	0	2	1	2	2	1	1	0	9
Cohen et al., 2018	2	2	0	1	1	1	2	2	11
De Tribolet-Hardy et al., 2024	0	2	1	1	2	2	2	0	10
Dombert et al., 2016	2	2	2	2	2	1	2	2	15
Klein et al., 2015	1	2	2	1	1	1	2	2	12
Koops et al., 2017	1	2	2	1	1	1	2	0	10
McPhail & Schmidt, 2023	0	2	2	2	1	2	2	0	11
Turner et al., 2016	1	2	2	1	1	1	2	0	10
Erkan et al., 2024	2	2	1	2	1	1	2	0	11
Geradt et al., 2018	0	2	1	2	1	0	2	1	9
Jahnke et al., 2015	0	2	1	2	2	2	2	2	13
Jahnke & Malon, 2018	2	2	1	2	1	2	2	2	14
Jahnke et al., 2023a	0	2	1	2	2	2	2	0	11
Jahnke et al., 2024a	0	2	1	2	1	2	2	0	10
Jahnke et al., 2024b	0	2	1	2	2	2	2	1	11
Jahnke et al., 2022b	2	2	1	2	2	1	2	2	14
Jahnke et al., 2023b	2	2	1	2	2	2	2	2	15
Jahnke et al., 2017	2	2	1	2	1	2	2	2	14
Jahnke et al., 2019	0	2	1	2	2	0	2	2	11
Konrad et al., 2017	2	2	0	2	2	0	2	2	12
Konrad et al., 2018	2	2	0	2	2	0	2	0	10
McPhail & Stephens, 2024	0	2	1	2	2	0	2	0	9
Mitchell & Galupo, 2015	2	2	1	2	2	1	1	0	11
Mitchell & Galupo, 2018a	2	2	1	2	1	1	2	0	11

Mitchell & Galupo, 2018b	2	2	1	2	0	1	2	0	10
Schaefer et al., 2010	2	2	1	2	2	1	2	0	12
Schuler et al., 2021	0	2	1	2	0	1	2	0	8
Stephens et al., 2023	2	2	1	2	2	0	2	2	13
Von Franque et al., 2023	0	2	1	2	2	1	2	0	10
Wilpert & Janssen, 2020	2	2	1	2	0	0	1	0	8
Inter-Rater Reliability (%)	79.4%	94.1%	67.6%	35.3%	67.6%	50%	82.3%	76.4%	

Note 1. Inter-rater reliability was reduced for Q4 due to a misalignment on the stringency of the requirements. Rater 1 only gave scores of 2 if there was a stated definition of CSA and CSAM in the introduction, whereas Rater 2 also gave scores of 2 if the definition was only embedded in the methodology. This was amended to favor Rater 2's interpretation.

Note 2. Inter-rater reliability was reduced for Q6 due to a misalignment regarding the absence/negligible amount of missing data. If there was no missing data or a negligible amount of missing data, Rater 1 gave scores of 2 irrespective of its acknowledgement, whereas Rater 2 only gave scores of 2 if it was acknowledged that there was no missing data, or the amount of data missing was negligible. This was amended to favor Rater 1's interpretation.

Note 3. For Q5, Rater 1 only gave scores of 2 if reliability/validity were described in the paper, whereas Rater 2 gave scores of 2 if the methods were reliable but not described in the paper. This was amended to favor Rater 2's interpretation.

Subgroup analysis – Pedohebephilic actors vs. non-actors

Variable	*k*	Model	Hedges' *g*	Lower CI	Upper CI	Lower PI	Upper PI	*p*-value	I2
Motivating Factors									
**Any Paraphilia**	**3**	**Fixed**	**0.27**	**0.13**	**0.4**	**-**	**-**	**<.001**	**0%**
		**Random**	**0.27**	**0.13**	**0.4**	**-0.62**	**1.15**	**<.001**	**0%**
Emotional Congruence with Children	2	Fixed	0.14	-0.12	0.39	-	-	.301	0%
		Random	0.14	-0.12	0.39	-1.53	1.81	.301	0%
Hypersexuality/Increased Sexual Desire	5	Fixed	0.00	-0.11	0.11	-	-	.974	35.55%
		Random	0.04	-0.18	0.25	-0.55	0.62	.735	45.27%
**Preferentiality for Children**	**11**	**Fixed**	**0.27**	**0.15**	**0.39**	**-**	**-**	**<.001**	**23.50%**
		**Random**	**0.25**	**0.11**	**0.39**	**-0.01**	**0.51**	**<.001**	**17.29%**
Preferentiality for Male Children	3	Fixed	-0.13	-0.37	0.1	-	-	.270	57.95%
		Random	-0.11	-0.47	0.26	-4	3.79	.572	57.29%
Facilitating Factors									
**Antisocial Behavior**	**5**	**Fixed**	**0.25**	**0.02**	**0.48**	**-**	**-**	**.035**	**63.14%**
		Random	0.26	-0.16	0.68	-1.14	1.65	.228	65.75%
**Cognitive Distortions**	**6**	**Fixed**	**0.27**	**0.14**	**0.41**	**-**	**-**	**<.001**	**25.57%**
		**Random**	**0.27**	**0.14**	**0.41**	**0.08**	**0.47**	**<.001**	**0.02%**
Empathy	3	Fixed	0.03	-0.27	0.32	-	-	.863	0%
		Random	0.03	-0.27	0.32	-1.86	1.92	.863	0%
Hostility	3	Fixed	0.21	-0.08	0.50	-	-	.155	0%
		Random	0.21	-0.08	0.50	-1.68	2.1	.155	0%
Impulsivity	2	Fixed	0.13	-0.07	0.33	-	-	.204	0%
		Random	0.13	-0.07	0.33	-1.17	1.43	.204	0%
**Lack of Control of Sexual Impulses**	**3**	**Fixed**	**0.60**	**0.32**	**0.89**	**-**	**-**	**<.001**	**0%**
		**Random**	**0.60**	**0.32**	**0.89**	**-1.23**	**2.44**	**<.001**	**0%**
Paranoid Ideation	3	Fixed	-0.02	-0.31	0.27	-	-	.904	0%
		Random	-0.02	-0.31	0.27	-1.91	1.87	.904	0%
Psychoticism	3	Fixed	0.17	-0.12	0.47	-	-	.240	0%
		Random	0.17	-0.12	0.47	-1.72	2.07	.240	0%

Substance Abuse	3	Fixed	0.23	-0.26	0.72	-	-	.364	0%
		Random	0.23	-0.26	0.72	-2.96	3.42	.364	0%
Situational Factors									
Contact With Children	2	Fixed	0.16	-0.11	0.43	-	-	0.237	88.29%
		Random	-0.01	-0.88	0.87	-9.42	9.41	0.991	88.29%
**Own Children**	**8**	**Fixed**	**0.35**	**0.18**	**0.53**	**-**	**-**	**<.001**	**0%**
		**Random**	**0.35**	**0.18**	**0.53**	**0.14**	**0.57**	**<.001**	**0%**
Other Factors									
**ACEs - Non-Sexual**	**4**	**Fixed**	**0.38**	**0.23**	**0.53**	**-**	**-**	**<.001**	**0%**
		**Random**	**0.38**	**0.23**	**0.53**	**0.05**	**0.71**	**<.001**	**0%**
**ACEs - Sexual**	**7**	**Fixed**	**0.46**	**0.34**	**0.59**	**-**	**-**	**<.001**	**45.06%**
		**Random**	**0.46**	**0.34**	**0.59**	**0.3**	**0.63**	**<.001**	**0.01%**
Affective Disorders	7	Fixed	0.05	-0.1	0.19	-	-	.543	0%
		Random	0.05	-0.1	0.19	-0.15	0.24	.543	0%
**Age**	**16**	**Fixed**	**0.41**	**0.34**	**0.49**	**-**	**-**	**<.001**	**79.63%**
		**Random**	**0.50**	**0.33**	**0.68**	**-0.13**	**1.13**	**<.001**	**76.64%**
Age of Onset	3	Fixed	-0.01	-0.14	0.12	-	-	.899	0%
		Random	-0.01	-0.14	0.12	-0.86	0.84	.898	0.03%
Agreeableness	2	Fixed	-0.24	-0.72	0.25	-	-	.338	0%
		Random	-0.24	-0.72	0.25	-3.36	2.89	.338	0%
Anxiety	6	Fixed	0.02	-0.13	0.17	-	-	.775	0%
		Random	0.02	-0.13	0.17	-0.19	0.24	.775	0%
Any Personality Disorder	2	Fixed	-0.20	-0.71	0.31	-	-	.439	0%
		Random	-0.20	-0.71	0.31	-3.48	3.08	.439	0%
Attention Deficit	2	Fixed	0.05	-0.64	0.73	-	-	.895	0%
		Random	0.05	-0.64	0.73	-4.38	4.47	.895	0%
Conscientiousness	2	Fixed	-0.05	-0.53	0.43	-	-	.831	0%
		Random	-0.05	-0.53	0.43	-3.18	3.07	.831	0%
Distress - General	5	Fixed	0.15	-0.03	0.34	-	-	.109	0%
		Random	0.15	-0.03	0.34	-0.15	0.46	.109	0%
**Distress - Sexual Interest**	**2**	**Fixed**	**0.20**	**0.10**	**0.30**	**-**	**-**	**<.001**	**3.46%**
		**Random**	**0.19**	**0.06**	**0.32**	**-0.86**	**1.24**	**.005**	**3.46%**

Education	12	Fixed	0.01	-0.09	0.11	-	-	.771	0%
		Random	0.01	-0.09	0.12	-0.11	0.14	.778	0.93%
Extraversion	2	Fixed	-0.12	-0.60	0.36	-	-	.619	0%
		Random	-0.12	-0.60	0.36	-3.25	3	.619	0%
**Head Injuries After Age 13**	**2**	**Fixed**	**0.34**	**0.07**	**0.61**	**-**	**-**	**.015**	**0%**
		**Random**	**0.34**	**0.07**	**0.61**	**-1.41**	**2.09**	**.015**	**0%**
Head Injuries Before Age 13	2	Fixed	0.02	-0.25	0.28	-	-	.900	0%
		Random	0.02	-0.25	0.28	-1.71	1.75	.900	0%
**Height**	**2**	**Fixed**	**-0.35**	**-0.62**	**-0.07**	**-**	**-**	**.013**	**24.92%**
		**Random**	**-0.34**	**-0.66**	**-0.02**	**-2.88**	**2.2**	**.037**	**24.92%**
**Intelligence**	**2**	**Fixed**	**-0.86**	**-1.18**	**-0.53**	**-**	**-**	**<.001**	**0%**
		**Random**	**-0.86**	**-1.18**	**-0.53**	**-2.95**	**1.24**	**<.001**	**0%**
Intimate Partner	7	Fixed	0.15	-0.01	0.31	-	-	.067	60.42%
		Random	0.10	-0.17	0.36	-0.66	0.85	.468	57.31%
Living Alone	2	Fixed	-0.12	-0.42	0.17	-	-	.414	0%
		Random	-0.12	-0.42	0.17	-2.06	1.81	.414	0%
Loneliness	3	Fixed	0.16	-0.1-	0.41	-	-	.232	60.84%
		Random	0.13	-0.32	0.58	-4.74	5	.572	62.12%
**Male Sex**	**7**	**Fixed**	**0.39**	**0.18**	**0.60**	**-**	**-**	**<.001**	**49.67%**
		**Random**	**0.44**	**0.09**	**0.80**	**-0.47**	**1.35**	**.014**	**48.54%**
Neuroticism	2	Fixed	-0.07	-0.55	0.41	-	-	.783	0%
		Random	-0.07	-0.55	0.41	-3.19	3.06	.783	0%
**Non-Heterosexuality**	**7**	**Fixed**	**0.21**	**0.08**	**0.33**	**-**	**-**	**.002**	**0%**
		**Random**	**0.20**	**0.06**	**0.34**	**-0.02**	**0.42**	**.005**	**5.61%**
**Non-Right Handedness**	**3**	**Fixed**	**0.19**	**0.03**	**0.35**	**-**	**-**	**.018**	**0%**
		**Random**	**0.19**	**0.03**	**0.35**	**-0.83**	**1.21**	**.018**	**0%**
Obsessive-Compulsive	3	Fixed	0.08	-0.21	0.37	-	-	.605	0%
		Random	0.08	-0.21	0.37	-1.81	1.97	.605	0%
Openness	2	Fixed	-0.31	-0.79	0.17	-	-	.208	37.24%
		Random	-0.45	-1.30	0.39	-8.15	7.24	.293	37.24%
Other Mental Health Diagnosis	4	Fixed	0.05	-0.20	0.30	-	-	.706	59.67%
		Random	0.04	-0.38	0.46	-1.6	1.69	.843	57.65%

Phobia	3	Fixed	0.13	-0.17	0.42	-	-	.401	0%
		Random	0.13	-0.17	0.42	-1.77	2.02	.401	0%
Self-Esteem	2	Fixed	0.10	-0.06	0.26	-	-	.205	0%
		Random	0.10	-0.06	0.26	-0.93	1.13	.205	0%
Social Desirability	4	Fixed	0.15	-0.08	0.37	-	-	.196	0%
		Random	0.15	-0.08	0.37	-0.35	0.64	.196	0%
Somatization	4	Fixed	0.04	-0.17	0.25	-	-	.724	52.79%
		Random	0.10	-0.23	0.43	-1.15	1.36	.538	52.34%
**Stigma**	**4**	**Fixed**	**0.61**	**0.53**	**0.68**	**-**	**-**	**<.001**	**97.15%**
		Random	0.21	-0.24	0.66	-1.91	2.33	.362	95.16%
Suicidality	3	Fixed	0.03	-0.20	0.25	-	-	.814	12.97%
		Random	0.04	-0.22	0.30	-2.03	2.11	.765	10.61%
**Therapy - Attendance**	**8**	**Fixed**	**0.28**	**0.14**	**0.43**	**-**	**-**	**<.001**	**73.81%**
		**Random**	**0.43**	**0.04**	**0.82**	**-0.79**	**1.66**	**.029**	**80.63%**
**Therapy - Interest**	**4**	**Fixed**	**0.43**	**0.26**	**0.59**	**-**	**-**	**<.001**	**91.39%**
		Random	0.45	-0.28	1.18	-3.02	3.92	.224	94.11%
Unemployment	5	Fixed	-0.01	-0.20	0.18	-	-	.917	19.09%
		Random	-0.01	-0.23	0.21	-0.51	0.49	.933	21.09%

Positive effect sizes indicate that a factor predicts acting, while negative effect sizes indicate that a factor predicts non-action.
Bolded factors are significant at the $p < .05$ level.

Subgroup analysis – CSAM actors vs. non-actors

Variable	*k*	Model	Hedges' *g*	Lower CI	Upper CI	Lower PI	Upper PI	*p*-value	I2
Motivating Factors									
**Any Paraphilia**	**4**	**Fixed**	**0.18**	**0.05**	**0.32**	**-**	**-**	**.008**	**54.04%**
		Random	0.01	-0.34	0.36	-1.3	1.32	.962	54.75%
Hypersexuality/Increased Sexual Desire	4	Fixed	-0.06	-0.19	0.06	-	-	.302	0%
		Random	-0.06	-0.19	0.06	-0.33	0.2	.302	0%
**Preferentiality for Children**	**2**	**Fixed**	**0.21**	**0.00**	**0.41**	**-**	**-**	**.049**	**49.79%**
		Random	0.05	-0.57	0.66	-5.95	6.05	.881	49.79%
**Preferentiality for Male Children**	**3**	**Fixed**	**0.34**	**0.13**	**0.54**	**-**	**-**	**.001**	**0%**
		**Random**	**0.33**	**0.09**	**0.56**	**-1.48**	**2.13**	**.006**	**10.55%**
Facilitating Factors									
Cognitive Distortions	2	Fixed	0.17	-0.04	0.38	-	-	.107	0%
		Random	0.17	-0.04	0.38	-1.19	1.54	.107	0%
Situational Factors									
Contact With Children	2	Fixed	0.21	0.03	0.39	-	-	.021	65.57%
		Random	0.13	-0.25	0.51	-3.62	3.88	.496	65.57%
**Own Children**	**4**	**Fixed**	**0.22**	**0.04**	**0.4**	**-**	**-**	**.015**	**0%**
		**Random**	**0.22**	**0.04**	**0.4**	**-0.17**	**0.62**	**.015**	**0%**
Other Factors									
**ACEs - Sexual**	**3**	**Fixed**	**0.36**	**0.22**	**0.5**	**-**	**-**	**<.001**	**68.39%**
		Random	0.28	-0.05	0.61	-3.56	4.11	.098	77.8%
Affective Disorders	3	Fixed	0.19	-0.17	0.56	-	-	.303	23.57%
		Random	0.19	-0.17	0.56	-2.18	2.57	.303	0%
**Age**	**7**	**Fixed**	**0.31**	**0.21**	**0.41**	**-**	**-**	**<.001**	**53.6%**
		**Random**	**0.41**	**0.23**	**0.6**	**-0.08**	**0.9**	**<.001**	**49.77%**
Anxiety	2	Fixed	0.24	-0.23	0.7	-	-	.319	0%
		Random	0.24	-0.23	0.7	-2.78	3.25	.319	0%
Any Personality Disorder	2	Fixed	-0.27	-0.79	0.26	-	-	.319	0%
		Random	-0.27	-0.79	0.26	-3.66	3.13	.319	0%

Distress - General	2	Fixed	0.06	-0.23	0.36	-	-	.668	0%
		Random	0.06	-0.23	0.36	-1.83	1.96	.668	0%
Education	4	Fixed	0.19	-0.04	0.42	-	-	.101	0%
		Random	0.19	-0.04	0.42	-0.31	0.7	.101	0%
Intimate Partner	3	Fixed	0.05	-0.23	0.32	-	-	.744	22.63%
		Random	0.07	-0.25	0.40	-2.67	2.81	.660	22%
**Male Sex**	**4**	**Fixed**	**0.52**	**0.23**	**0.81**	**-**	**-**	**<.001**	**0%**
		**Random**	**0.52**	**0.23**	**0.81**	**-0.12**	**1.16**	**<.001**	**0%**
**Non-Heterosexuality**	**2**	**Fixed**	**0.26**	**0.09**	**0.43**	**-**	**-**	**.002**	**0%**
		**Random**	**0.26**	**0.09**	**0.43**	**-0.83**	**1.36**	**.002**	**0%**
Unemployment	3	Fixed	0.00	-0.36	0.37	-	-	.983	64.99%
		Random	0.08	-0.56	0.72	-7.05	7.21	.803	65.31%

Positive effect sizes indicate that a factor predicts acting, while negative effect sizes indicate that a factor predicts non-action.
Bolded factors are significant at the $p < .05$ level.

Subgroup analysis – CSA actors vs. non-Actors

Variable	*k*	Model	Hedges' *g*	Lower CI	Upper CI	Lower PI	Upper PI	*p*-value	I2
Motivating Factors									
Any Paraphilia	3	Fixed	-0.11	-0.61	0.38	-	-	.651	0.84%
		Random	-0.11	-0.61	0.38	-3.32	3.09	.651	0%
Hypersexuality/Increased Sexual Desire	3	Fixed	-0.01	-0.42	0.41	-	-	.968	4.52%
		Random	0.01	-0.48	0.50	-3.95	3.97	.976	15.36%
**Preferentiality for Children**	**2**	**Fixed**	**0.22**	**0.00**	**0.43**	**-**	**-**	**.048**	**59.33%**
		Random	0.42	-0.28	1.13	-6.63	7.48	.240	59.33%
**Preferentiality for Male Children**	**2**	**Fixed**	**0.27**	**0.05**	**0.50**	**-**	**-**	**.018**	**0%**
		**Random**	**0.27**	**0.05**	**0.50**	**-1.2**	**1.74**	**.018**	**0%**
Facilitating Factors									
Cognitive Distortions	2	Fixed	0.08	-0.13	0.29	-	-	.440	0%
		Random	0.08	-0.13	0.29	-1.28	1.45	.440	0%
Situational Factors									
Contact With Children	**2**	Fixed	0.24	0.04	0.44	-	-	.018	54.96%
		Random	0.00	-0.81	0.82	-8.08	8.09	.991	54.96%
**Own Children**	**4**	**Fixed**	**0.28**	**0.11**	**0.45**	**-**	**-**	**.002**	**22.21%**
		**Random**	**0.28**	**0.09**	**0.47**	**-0.19**	**0.76**	**.004**	**3.08%**
Other Factors									
**ACEs - Sexual**	**2**	**Fixed**	**0.27**	**0.09**	**0.46**	**-**	**-**	**.004**	**0%**
		**Random**	**0.27**	**0.09**	**0.46**	**-0.93**	**1.48**	**.004**	**0%**
Affective Disorders	2	Fixed	0.09	-0.35	0.53	-	-	.685	0%
		Random	0.09	-0.35	0.53	-2.77	2.95	.685	0%
**Age**	**6**	**Fixed**	**0.56**	**0.41**	**0.71**	**-**	**-**	**<.001**	**36.82%**
		**Random**	**0.61**	**0.38**	**0.84**	**0.02**	**1.2**	**<.001**	**39.65%**
Distress - General	2	Fixed	0.06	-0.34	0.45	-	-	.773	0%
		Random	0.06	-0.34	0.45	-2.51	2.62	.773	0%
Education	6	Fixed	0.01	-0.18	0.21	-	-	.882	0%
		Random	0.01	-0.18	0.21	-0.26	0.29	.882	0%

Intimate Partner	3	Fixed	0.02	-0.38	0.42	-	-	.934	65.65%
		Random	0.02	-0.75	0.79	-8.72	8.76	.958	68.92%
Male Sex	2	Fixed	0.71	-0.47	1.9	-	-	.237	0%
		Random	0.71	-0.47	1.9	-6.97	8.4	.237	0%
Non-Heterosexuality	2	Fixed	0.33	-0.26	0.91	-	-	.272	0%
		Random	0.33	-0.26	0.91	-3.44	4.1	.272	0%
**Therapy - Attendance**	**2**	**Fixed**	**0.77**	**0.08**	**1.47**	**-**	**-**	**.029**	**89.12%**
		Random	1.05	-1.15	3.24	-22.69	24.79	.349	89.12%
**Unemployment**	**3**	**Fixed**	**0.54**	**0.12**	**0.96**	**-**	**-**	**.011**	**31.9%**
		**Random**	**0.54**	**0.10**	**0.98**	**-2.6**	**3.68**	**.015**	**6.43%**

Positive effect sizes indicate that a factor predicts acting, while negative effect sizes indicate that a factor predicts non-action
Bolded factors are significant at the p < .05 level.

Appendix F. Funnel Plot Asymmetry

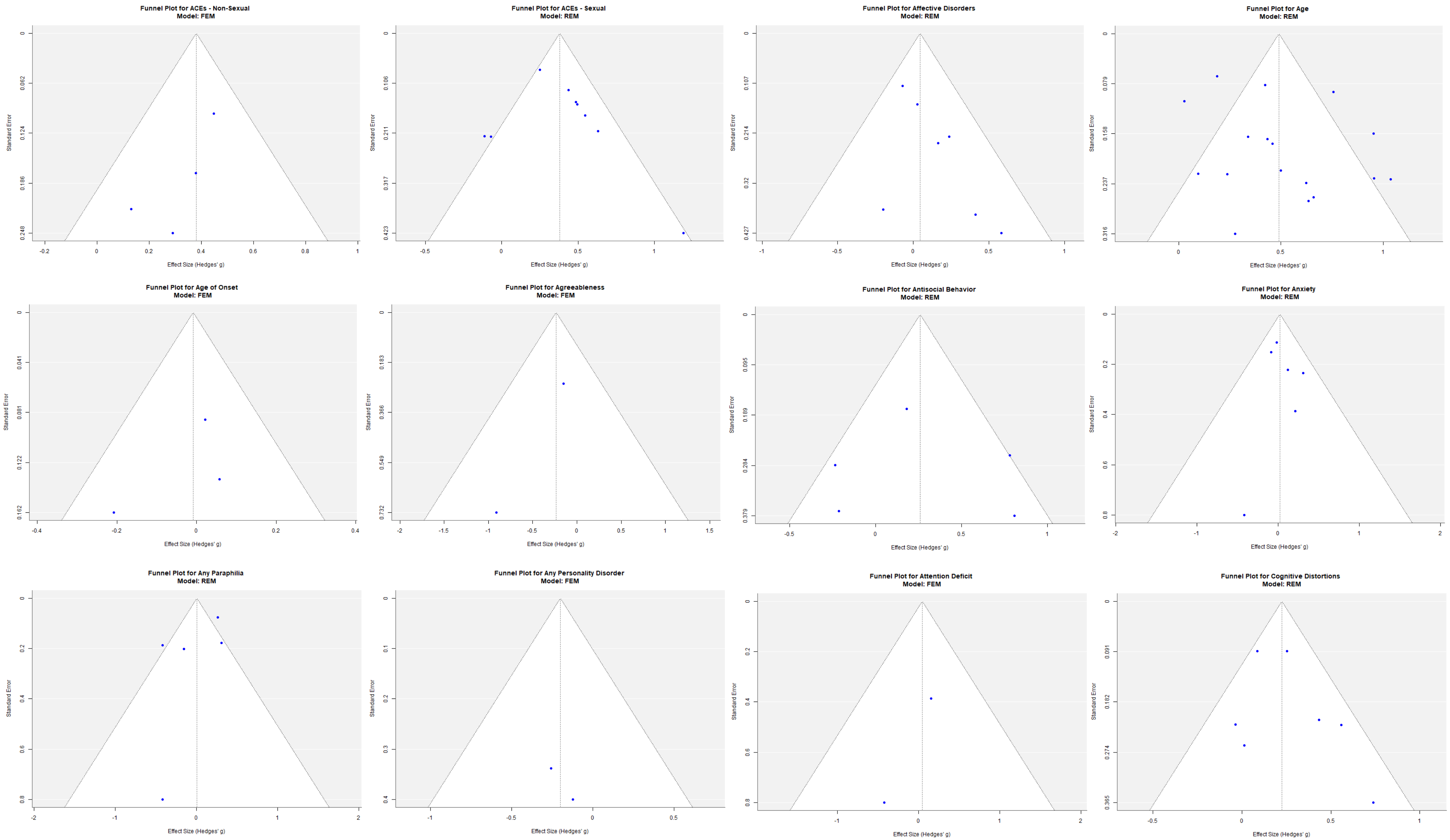

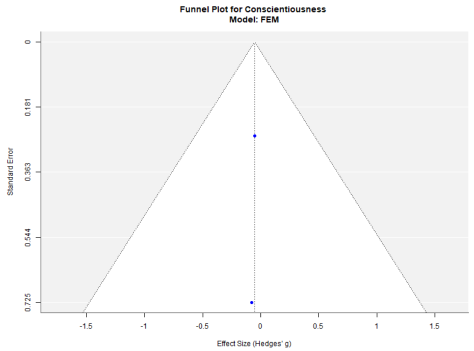
Funnel Plot for Conscientiousness
Model: FEM
Standard Error
0
0.181
0.363
0.544
0.725
-1.5
-1
-0.5
0
0.5
1
1.5
Effect Size (Hedges' g)

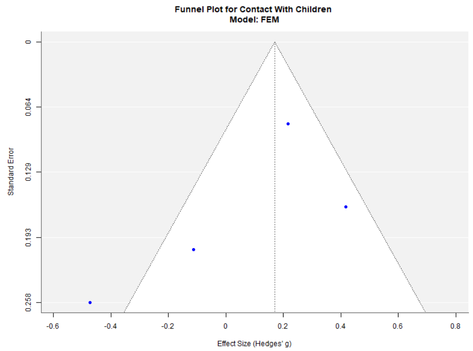
Funnel Plot for Contact With Children
Model: FEM
Standard Error
0
0.064
0.129
0.193
0.258
-0.6
-0.4
-0.2
0
0.2
0.4
0.6
0.8
Effect Size (Hedges' g)

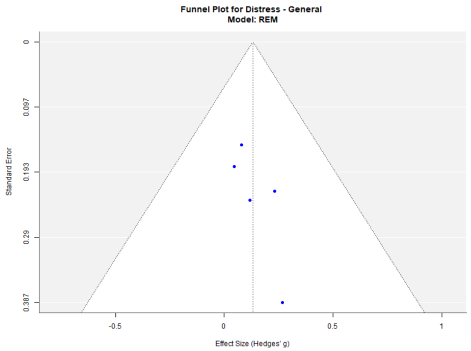
Funnel Plot for Distress - General
Model: REM
Standard Error
0
0.097
0.193
0.29
0.387
-0.5
0
0.5
1
Effect Size (Hedges' g)

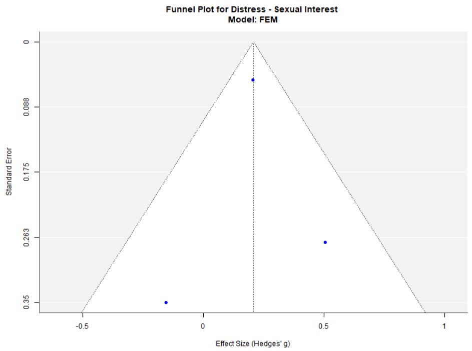
Funnel Plot for Distress - Sexual Interest
Model: FEM
Standard Error
0
0.088
0.175
0.263
0.35
-0.5
0
0.5
1
Effect Size (Hedges' g)

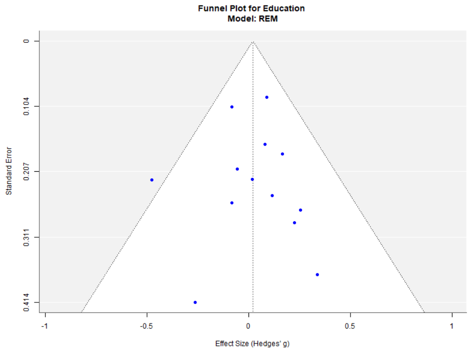
Funnel Plot for Education
Model: REM
Standard Error
0
0.104
0.207
0.311
0.414
-1
-0.5
0
0.5
1
Effect Size (Hedges' g)

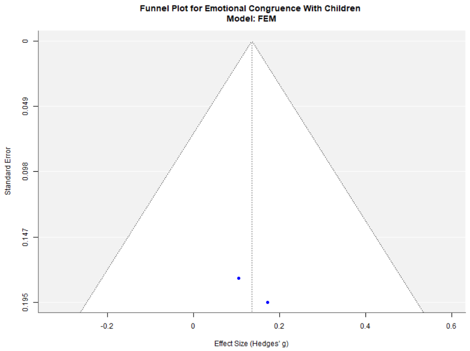
Funnel Plot for Emotional Congruence With Children
Model: FEM
Standard Error
0
0.049
0.098
0.147
0.195
-0.2
0
0.2
0.4
0.6
Effect Size (Hedges' g)

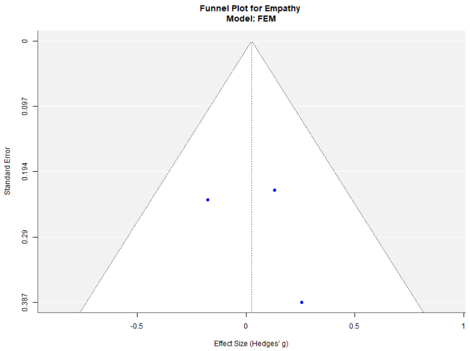
Funnel Plot for Empathy
Model: FEM
Standard Error
0
0.097
0.194
0.29
0.387
-0.5
0
0.5
1
Effect Size (Hedges' g)

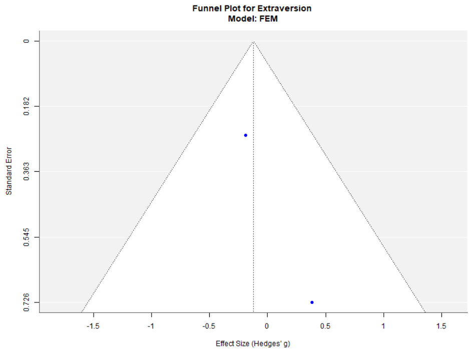
Funnel Plot for Extraversion
Model: FEM
Standard Error
0
0.182
0.363
0.545
0.726
-1.5
-1
-0.5
0
0.5
1
1.5
Effect Size (Hedges' g)

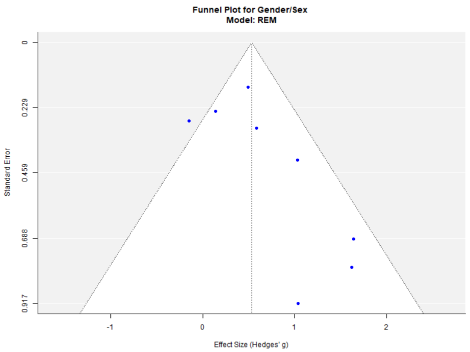
Funnel Plot for Gender/Sex
Model: REM
Standard Error
0
0.229
0.459
0.688
0.917
-1
0
1
2
Effect Size (Hedges' g)

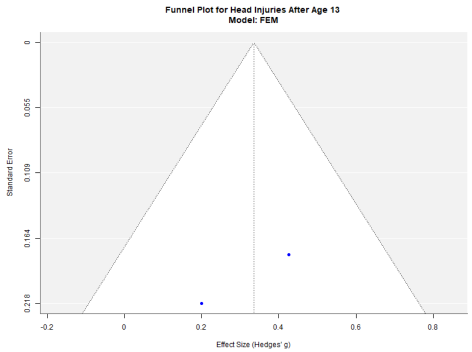
Funnel Plot for Head Injuries After Age 13
Model: FEM
Standard Error
0
0.055
0.109
0.164
0.218
-0.2
0
0.2
0.4
0.6
0.8
Effect Size (Hedges' g)

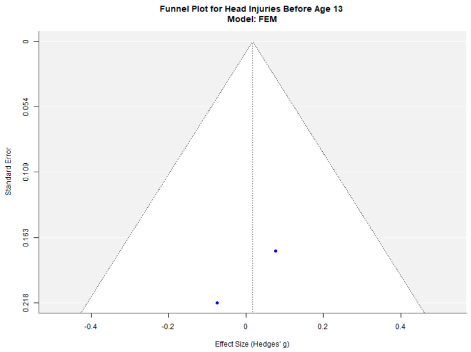
Funnel Plot for Head Injuries Before Age 13
Model: FEM
Standard Error
0
0.054
0.109
0.163
0.218
-0.4
-0.2
0
0.2
0.4
Effect Size (Hedges' g)

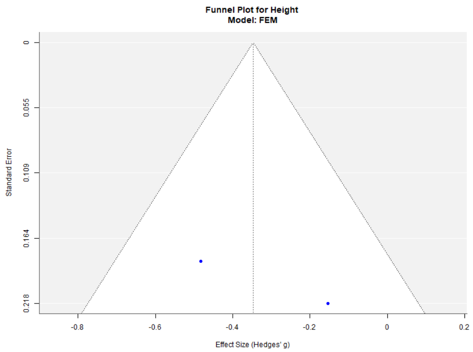
Funnel Plot for Height
Model: FEM
Standard Error
0
0.055
0.109
0.164
0.218
-0.8
-0.6
-0.4
-0.2
0
0.2
Effect Size (Hedges' g)

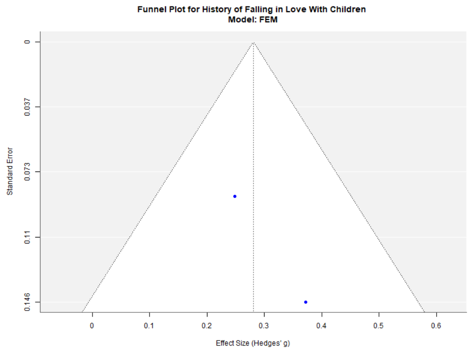
Funnel Plot for History of Falling in Love With Children
Model: FEM
Standard Error
0
0.037
0.073
0.11
0.146
0
0.1
0.2
0.3
0.4
0.5
0.6
Effect Size (Hedges' g)

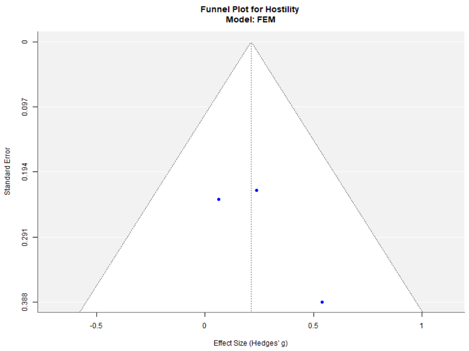
Funnel Plot for Hostility
Model: FEM
Standard Error
0
0.097
0.194
0.291
0.388
-0.5
0
0.5
1
Effect Size (Hedges' g)

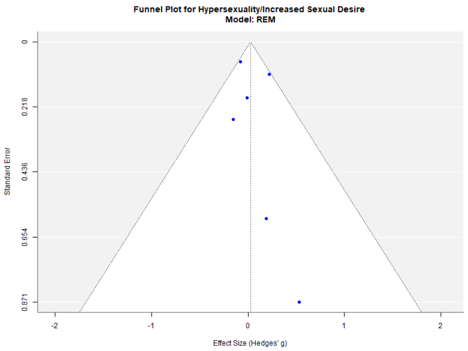
Funnel Plot for Hypersexuality/Increased Sexual Desire
Model: REM
Standard Error
0
0.218
0.436
0.654
0.871
-2
-1
0
1
2
Effect Size (Hedges' g)

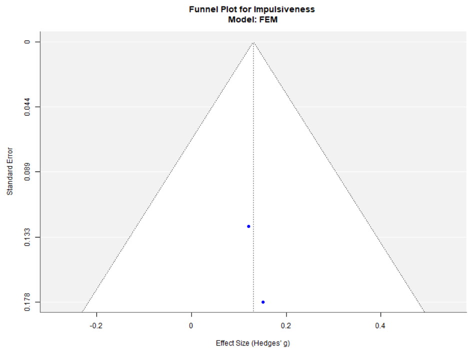
Funnel Plot for Impulsiveness
Model: FEM
Standard Error
0
0.044
0.089
0.133
0.178
-0.2
0
0.2
0.4
Effect Size (Hedges' g)

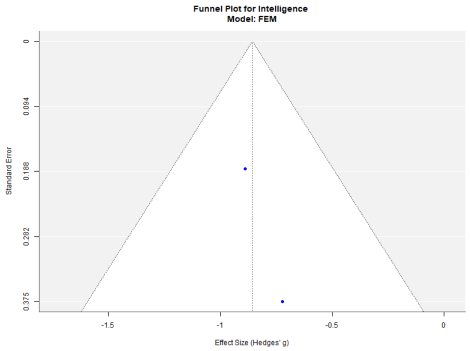
Funnel Plot for Intelligence
Model: FEM
Standard Error
0
0.094
0.188
0.282
0.375
-1.5
-1
-0.5
0
Effect Size (Hedges' g)

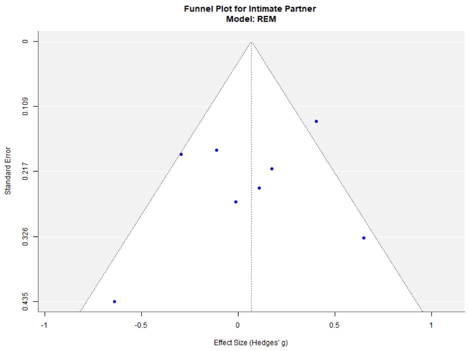
Funnel Plot for Intimate Partner
Model: REM
Standard Error
0
0.109
0.217
0.326
0.435
-1
-0.5
0
0.5
1
Effect Size (Hedges' g)

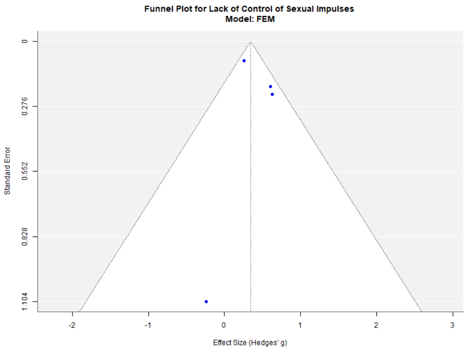
Funnel Plot for Lack of Control of Sexual Impulses
Model: FEM
Standard Error
0
0.276
0.552
0.828
1.104
-2
-1
0
1
2
3
Effect Size (Hedges' g)

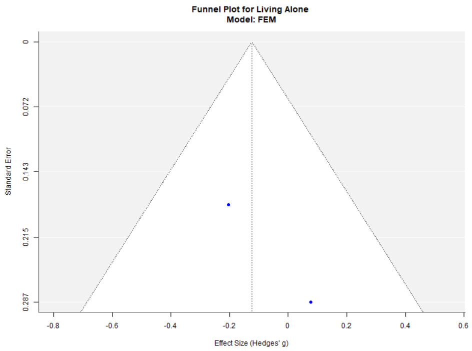
Funnel Plot for Living Alone
Model: FEM
Standard Error
0
0.072
0.143
0.215
0.287
-0.8
-0.6
-0.4
-0.2
0
0.2
0.4
0.6
Effect Size (Hedges' g)

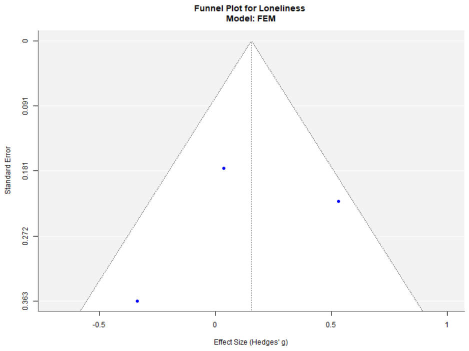
Funnel Plot for Loneliness
Model: FEM
Standard Error
0
0.091
0.181
0.272
0.363
-0.5
0
0.5
1
Effect Size (Hedges' g)

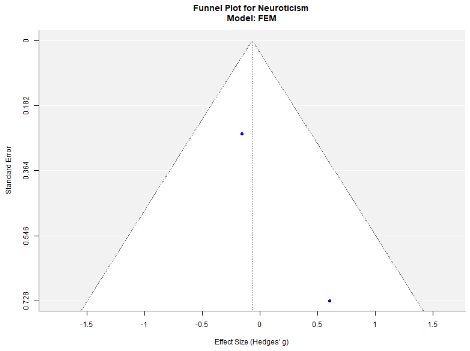
Funnel Plot for Neuroticism
Model: FEM
Standard Error
0
0.182
0.364
0.546
0.728
-1.5
-1
-0.5
0
0.5
1
1.5
Effect Size (Hedges' g)

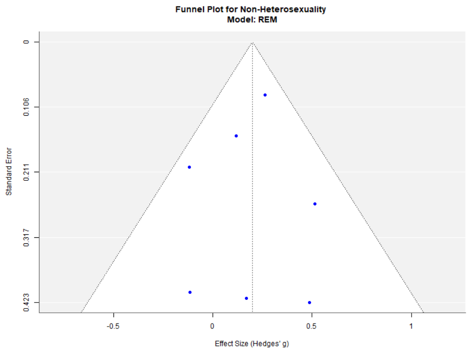
Funnel Plot for Non-Heterosexuality
Model: REM
Standard Error
0
0.106
0.211
0.317
0.423
-0.5
0
0.5
1
Effect Size (Hedges' g)

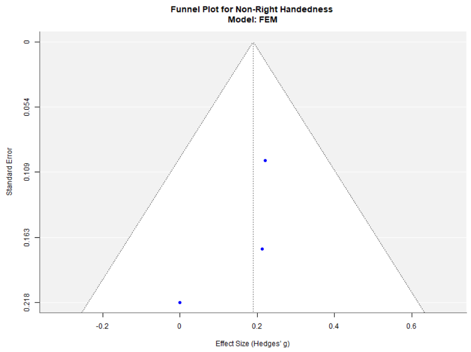
Funnel Plot for Non-Right Handedness
Model: FEM
Standard Error
0
0.054
0.109
0.163
0.218
-0.2
0
0.2
0.4
0.6
Effect Size (Hedges' g)

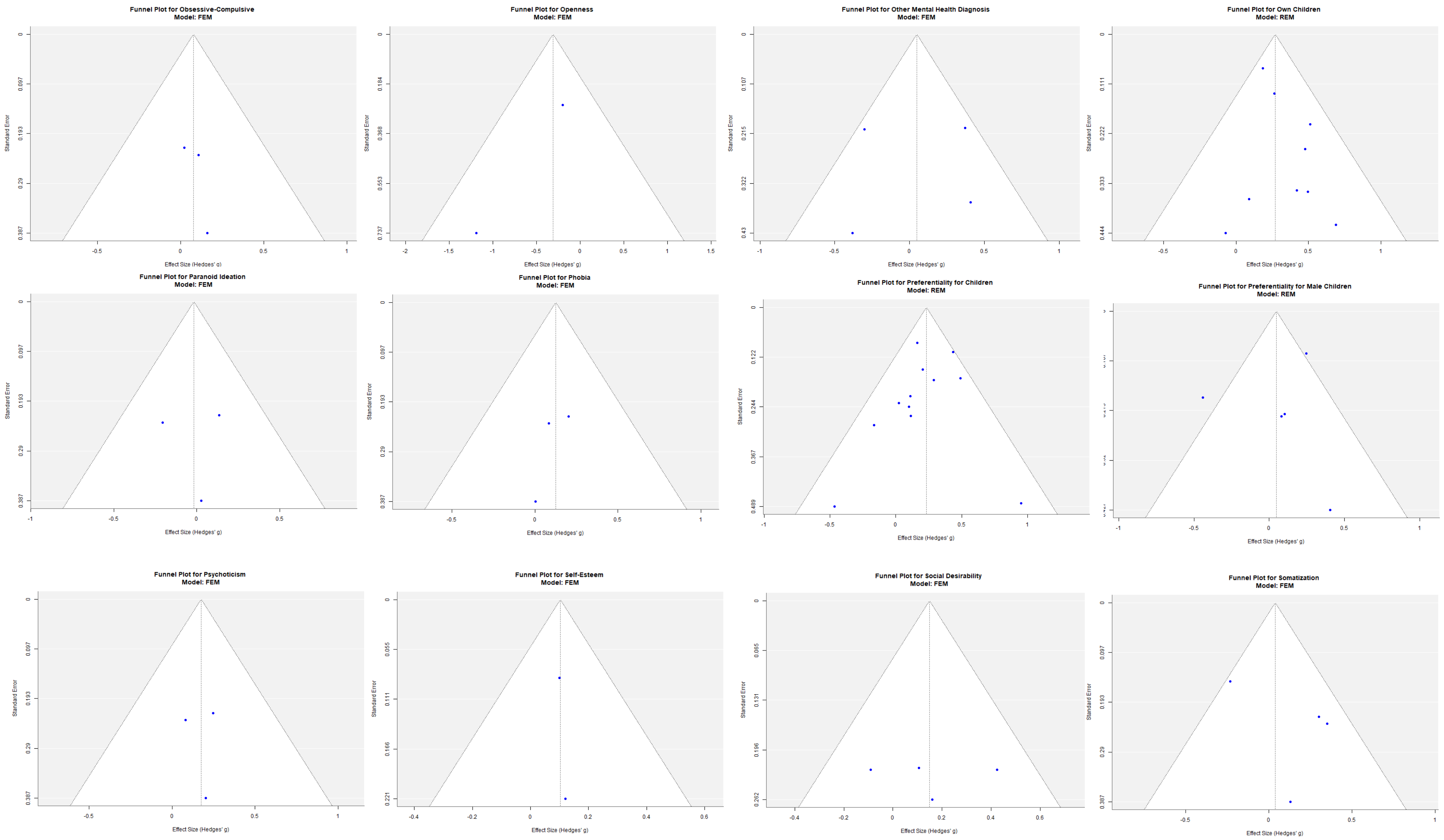
Funnel Plot for Obsessive-Compulsive
Model: FEM
Funnel Plot for Openness
Model: FEM
Funnel Plot for Other Mental Health Diagnosis
Model: FEM
Funnel Plot for Own Children
Model: REM
Funnel Plot for Paranoid Ideation
Model: FEM
Funnel Plot for Phobia
Model: FEM
Funnel Plot for Preferentiality for Children
Model: REM
Funnel Plot for Preferentiality for Male Children
Model: REM
Funnel Plot for Psychoticism
Model: FEM
Funnel Plot for Self-Esteem
Model: FEM
Funnel Plot for Social Desirability
Model: FEM
Funnel Plot for Somatization
Model: FEM
Standard Error
Effect Size (Hedges' g)

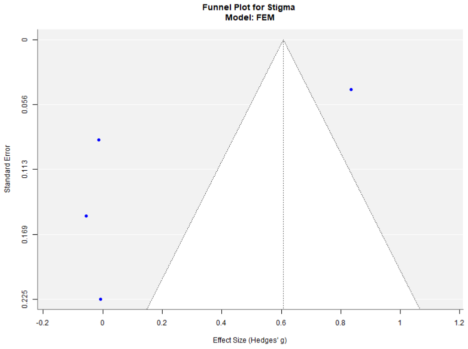
Funnel Plot for Stigma
Model: FEM
Standard Error
Effect Size (Hedges' g)

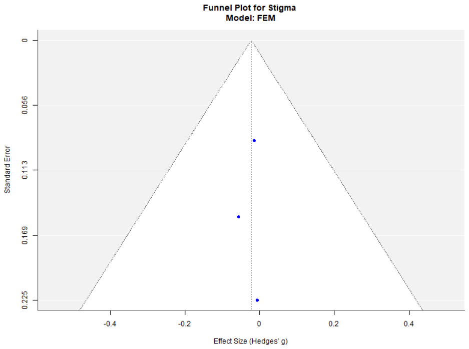
Funnel Plot for Stigma
Model: FEM
Standard Error
Effect Size (Hedges' g)

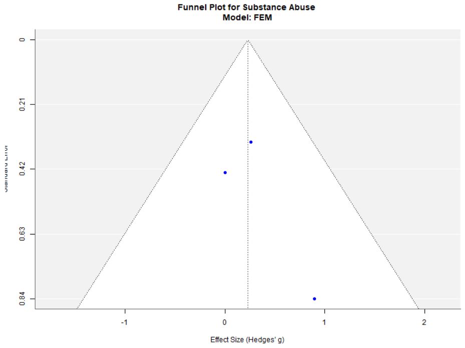
Funnel Plot for Substance Abuse
Model: FEM
Effect Size (Hedges' g)

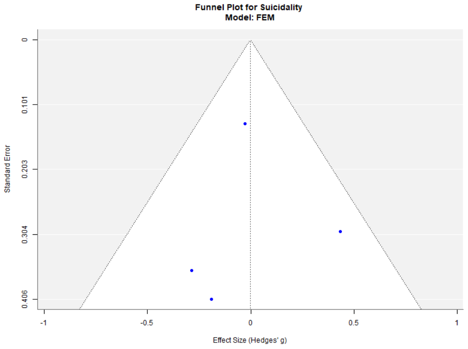
Funnel Plot for Suicidality
Model: FEM
Standard Error
Effect Size (Hedges' g)

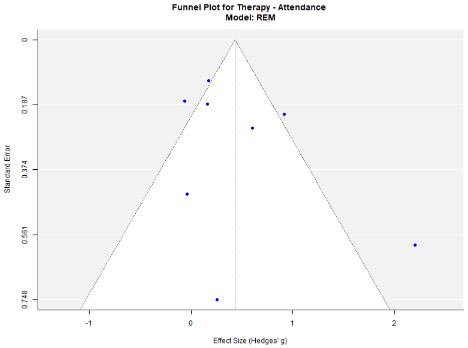
Funnel Plot for Therapy - Attendance
Model: REM
Standard Error
Effect Size (Hedges' g)

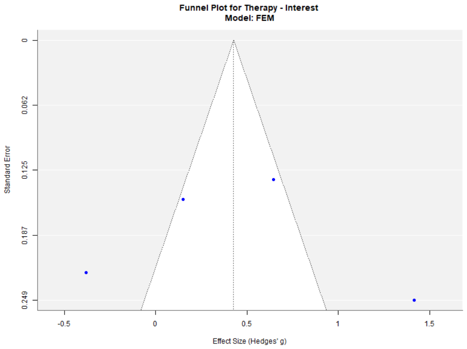
Funnel Plot for Therapy - Interest
Model: FEM
Standard Error
Effect Size (Hedges' g)

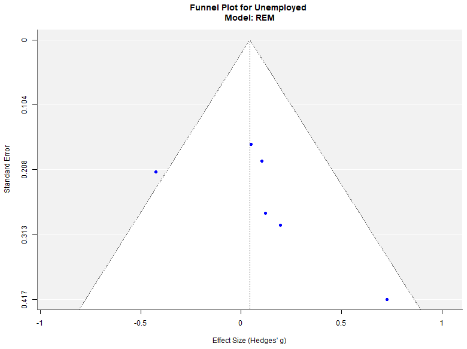
Funnel Plot for Unemployed
Model: REM
Standard Error
Effect Size (Hedges' g)

Egger's Test

**Variable**	***N***	**Model**	**Hedges' *g***	**Lower CI**	**Upper CI**	***p*-value**	**Egger's *p***
Motivating Factors							
Any Paraphilia	5	REM	0.00	-0.32	0.33	.981	-.936
Hypersexuality/Increased Sexual Desire	6	REM	0.032	-0.15	0.20	.759	.400
Intimate Partner	7	REM	-0.06	-0.21	0.33	.666	-0.694
Preferentiality for Children	12	REM	0.23	0.12	0.35	<.001	-.921
Preferentiality for Male Children	5	REM	0.05	-0.24	0.33	.748	.262
Facilitating Factors							
Antisocial Behavior	5	REM	0.26	-0.16	0.68	.228	.094
Cognitive Distortions	7	REM	0.22	0.08	0.37	.003	1.068
Situational Factors							
Own Children	9	REM	0.27	0.15	0.39	<.001	1.222
Other Factors							
ACEs - Sexual	9	REM	0.38	0.22	0.54	<.001	.864
Affective Disorders	6	REM	0.06	-0.09	0.22	.438	1.539
Age	18	REM	0.49	0.34	0.64	<.001	1.018
Anxiety	6	REM	0.02	-0.13	0.17	.775	.469
Distress – General	6	REM	0.13	-0.04	0.30	.124	.642
Education	13	REM	0.02	-0.07	0.12	.674	.030
Male Sex	7	REM	0.61	0.28	0.93	<.001	2.145
Non-Heterosexuality	7	REM	0.20	0.06	0.34	.005	-.164
Therapy – Attendance	8	REM	0.43	0.04	0.82	.029	1.317
Therapy – Attendance (Without Outlier)	5	REM	0.11	-0.06	0.27	.195	-.282
Unemployment	6	REM	0.04	-0.19	0.27	.713	1.444

Positive effect sizes indicate that a factor predicts acting, while negative effect sizes indicate that a factor predicts non-action.
Bolded factors are significant at the $p < .05$ level.

Forest Plots

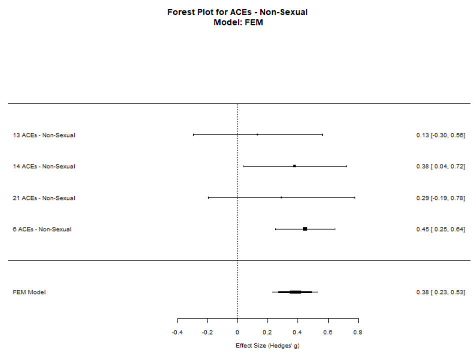

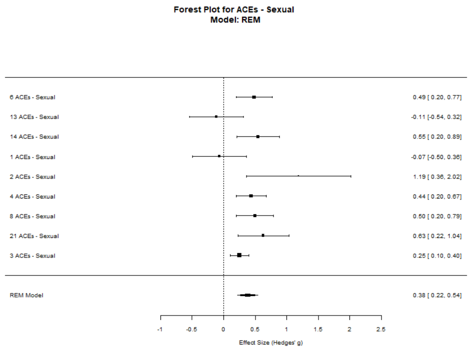

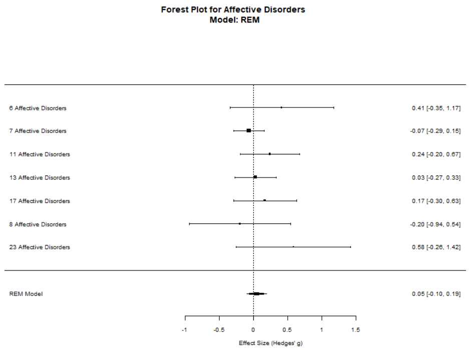

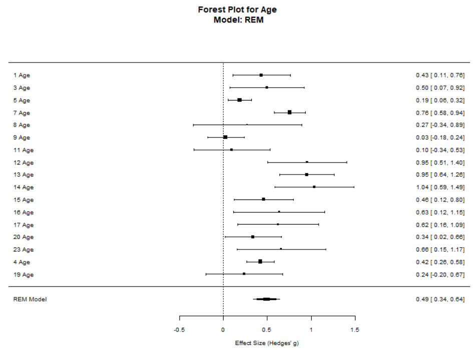

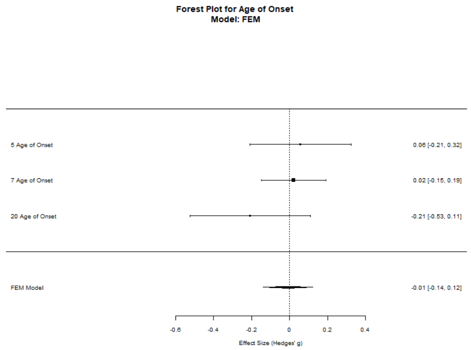

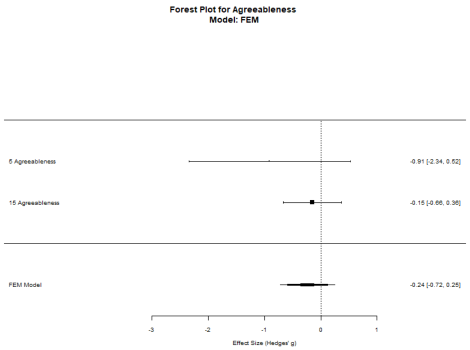

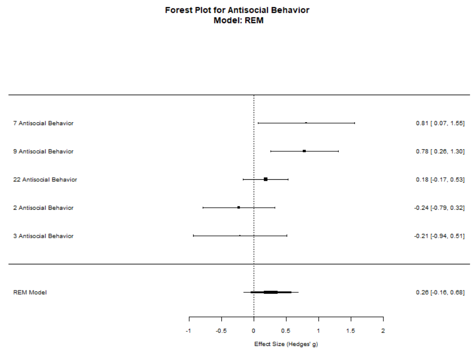

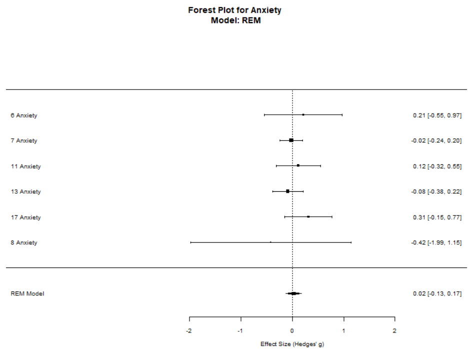

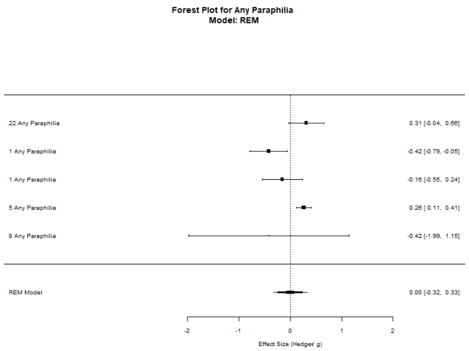

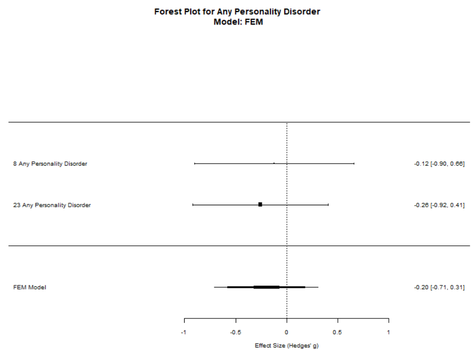
Forest Plot for Any Personality Disorder
Model: FEM
8 Any Personality Disorder
-0.12 [-0.90, 0.66]
23 Any Personality Disorder
-0.26 [-0.92, 0.41]
FEM Model
-0.20 [-0.71, 0.31]
Effect Size (Hedges' g)

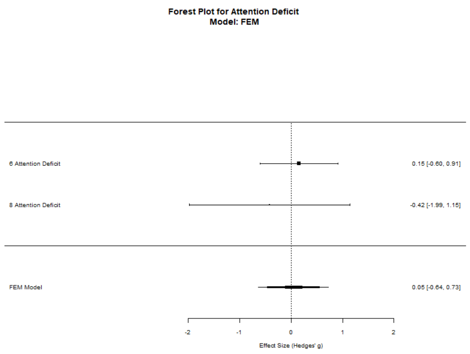
Forest Plot for Attention Deficit
Model: FEM
6 Attention Deficit
0.15 [-0.60, 0.91]
8 Attention Deficit
-0.42 [-1.99, 1.16]
FEM Model
0.05 [-0.64, 0.73]
Effect Size (Hedges' g)

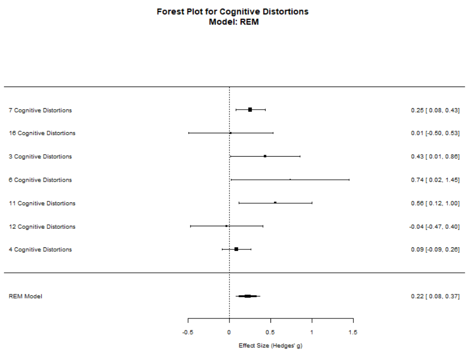
Forest Plot for Cognitive Distortions
Model: REM
7 Cognitive Distortions
0.25 [0.08, 0.43]
16 Cognitive Distortions
0.01 [-0.50, 0.53]
3 Cognitive Distortions
0.43 [0.01, 0.86]
6 Cognitive Distortions
0.74 [0.02, 1.45]
11 Cognitive Distortions
0.56 [0.12, 1.00]
12 Cognitive Distortions
-0.04 [-0.47, 0.40]
4 Cognitive Distortions
0.09 [-0.09, 0.26]
REM Model
0.22 [0.08, 0.37]
Effect Size (Hedges' g)

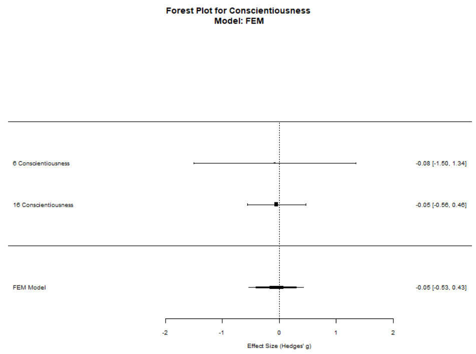
Forest Plot for Conscientiousness
Model: FEM
6 Conscientiousness
-0.08 [-1.50, 1.34]
16 Conscientiousness
-0.05 [-0.56, 0.46]
FEM Model
-0.05 [-0.53, 0.43]
Effect Size (Hedges' g)

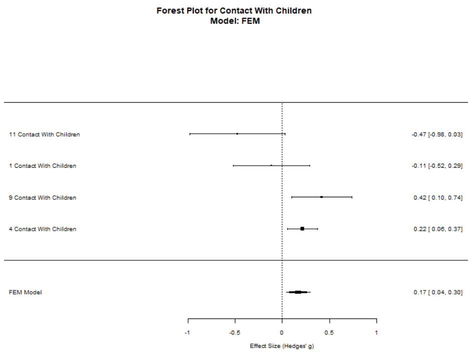
Forest Plot for Contact With Children
Model: FEM
11 Contact With Children
-0.47 [-0.98, 0.03]
1 Contact With Children
-0.11 [-0.52, 0.29]
9 Contact With Children
0.42 [0.10, 0.74]
4 Contact With Children
0.22 [0.06, 0.37]
FEM Model
0.17 [0.04, 0.30]
Effect Size (Hedges' g)

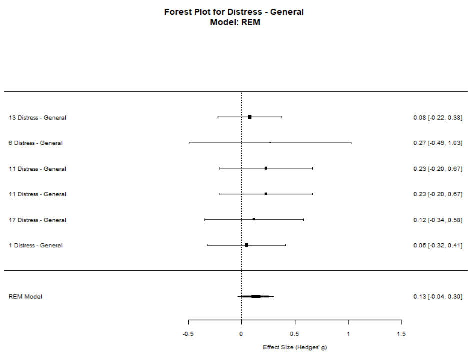
Forest Plot for Distress - General
Model: REM
13 Distress - General
0.08 [-0.22, 0.38]
6 Distress - General
0.27 [-0.49, 1.03]
11 Distress - General
0.23 [-0.20, 0.67]
11 Distress - General
0.23 [-0.20, 0.67]
17 Distress - General
0.12 [-0.34, 0.58]
1 Distress - General
0.05 [-0.32, 0.41]
REM Model
0.13 [-0.04, 0.30]
Effect Size (Hedges' g)

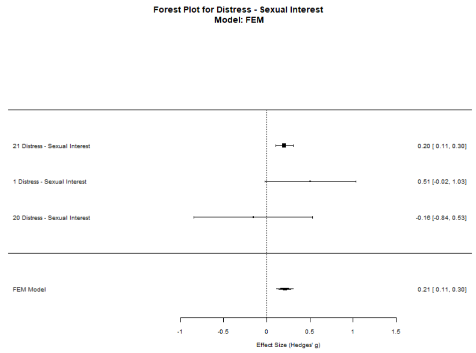
Forest Plot for Distress - Sexual Interest
Model: FEM
21 Distress - Sexual Interest
0.20 [0.11, 0.30]
1 Distress - Sexual Interest
0.51 [-0.02, 1.03]
20 Distress - Sexual Interest
-0.16 [-0.84, 0.53]
FEM Model
0.21 [0.11, 0.30]
Effect Size (Hedges' g)

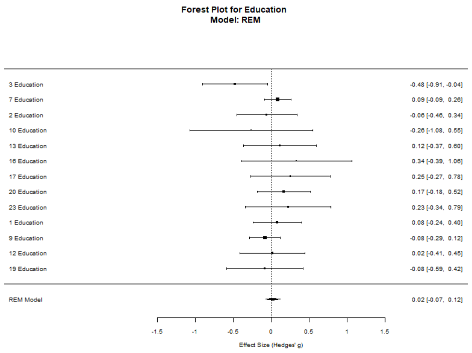
Forest Plot for Education
Model: REM
3 Education
-0.48 [-0.91, -0.04]
7 Education
0.09 [-0.09, 0.26]
2 Education
-0.06 [-0.46, 0.34]
10 Education
-0.26 [-1.08, 0.55]
13 Education
0.12 [-0.37, 0.60]
16 Education
0.34 [-0.39, 1.06]
17 Education
0.25 [-0.27, 0.78]
20 Education
0.17 [-0.18, 0.52]
23 Education
0.23 [-0.34, 0.79]
1 Education
0.08 [-0.24, 0.40]
9 Education
-0.08 [-0.29, 0.12]
12 Education
0.02 [-0.41, 0.45]
19 Education
-0.08 [-0.59, 0.42]
REM Model
0.02 [-0.07, 0.12]
Effect Size (Hedges' g)

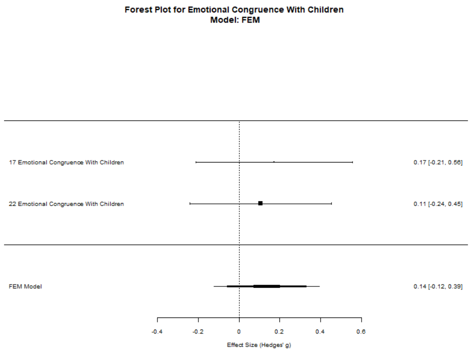
Forest Plot for Emotional Congruence With Children
Model: FEM
17 Emotional Congruence With Children
0.17 [-0.21, 0.56]
22 Emotional Congruence With Children
0.11 [-0.24, 0.46]
FEM Model
0.14 [-0.12, 0.39]
Effect Size (Hedges' g)

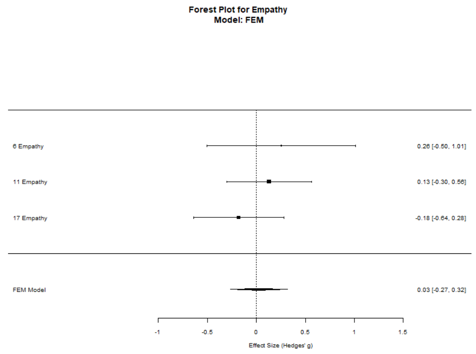
Forest Plot for Empathy
Model: FEM
6 Empathy
0.26 [-0.50, 1.01]
11 Empathy
0.13 [-0.30, 0.56]
17 Empathy
-0.18 [-0.64, 0.28]
FEM Model
0.03 [-0.27, 0.32]
-1 -0.5 0 0.5 1 1.5
Effect Size (Hedges' g)

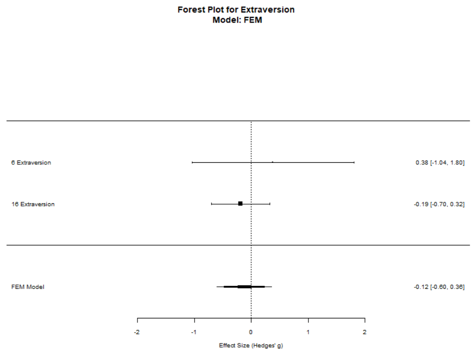
Forest Plot for Extraversion
Model: FEM
6 Extraversion
0.38 [-1.04, 1.80]
16 Extraversion
-0.19 [-0.70, 0.32]
FEM Model
-0.12 [-0.60, 0.36]
-2 -1 0 1 2
Effect Size (Hedges' g)

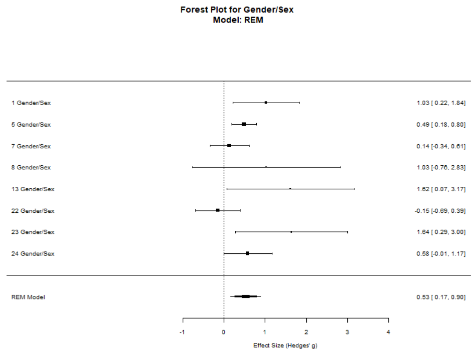
Forest Plot for Gender/Sex
Model: REM
1 Gender/Sex
1.03 [0.22, 1.84]
5 Gender/Sex
0.49 [0.18, 0.80]
7 Gender/Sex
0.14 [-0.34, 0.61]
8 Gender/Sex
1.03 [-0.76, 2.83]
13 Gender/Sex
1.62 [0.07, 3.17]
22 Gender/Sex
-0.15 [-0.69, 0.39]
23 Gender/Sex
1.64 [0.29, 3.00]
24 Gender/Sex
0.58 [-0.01, 1.17]
REM Model
0.53 [0.17, 0.90]
-1 0 1 2 3 4
Effect Size (Hedges' g)

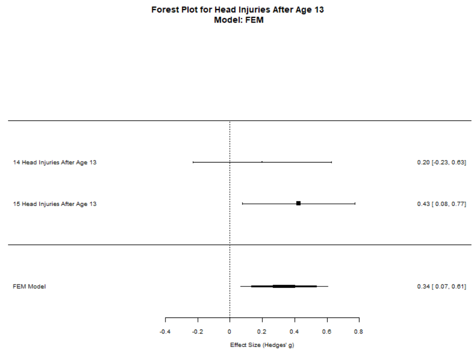
Forest Plot for Head Injuries After Age 13
Model: FEM
14 Head Injuries After Age 13
0.20 [-0.23, 0.63]
15 Head Injuries After Age 13
0.43 [0.08, 0.77]
FEM Model
0.34 [0.07, 0.61]
-0.4 -0.2 0 0.2 0.4 0.6 0.8
Effect Size (Hedges' g)

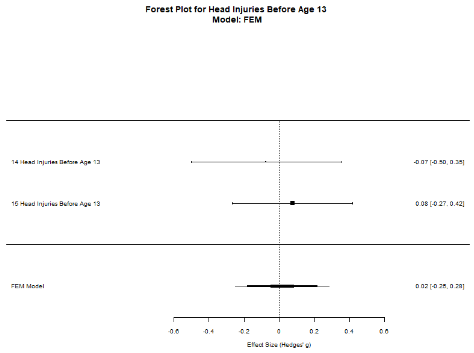
Forest Plot for Head Injuries Before Age 13
Model: FEM
14 Head Injuries Before Age 13
-0.07 [-0.50, 0.35]
15 Head Injuries Before Age 13
0.08 [-0.27, 0.42]
FEM Model
0.02 [-0.25, 0.28]
-0.6 -0.4 -0.2 0 0.2 0.4 0.6
Effect Size (Hedges' g)

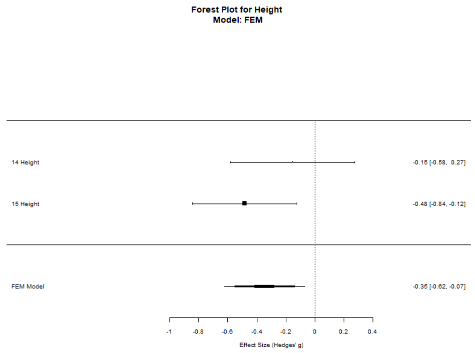
Forest Plot for Height
Model: FEM
14 Height
-0.15 [-0.58, 0.27]
15 Height
-0.48 [-0.84, -0.12]
FEM Model
-0.35 [-0.62, -0.07]
-1 -0.8 -0.6 -0.4 -0.2 0 0.2 0.4
Effect Size (Hedges' g)

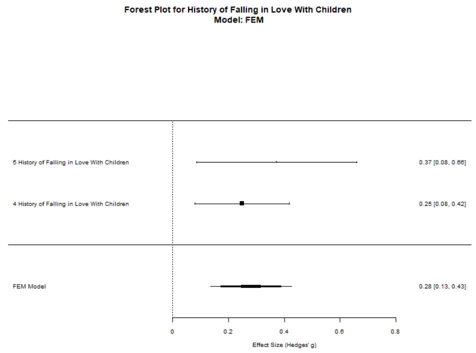
Forest Plot for History of Falling in Love With Children
Model: FEM
5 History of Falling in Love With Children
0.37 [0.08, 0.66]
4 History of Falling in Love With Children
0.25 [0.08, 0.42]
FEM Model
0.28 [0.13, 0.43]
0 0.2 0.4 0.6 0.8
Effect Size (Hedges' g)

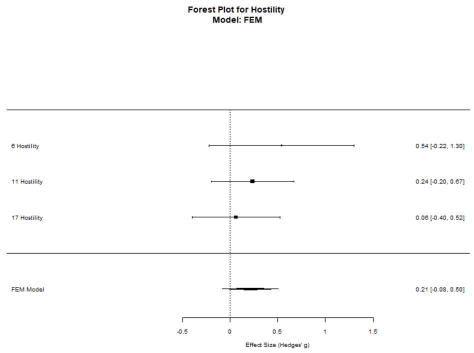
Forest Plot for Hostility
Model: FEM
6 Hostility
0.54 [-0.22, 1.30]
11 Hostility
0.24 [-0.20, 0.67]
17 Hostility
0.06 [-0.40, 0.52]
FEM Model
0.21 [-0.08, 0.50]
-0.5 0 0.5 1 1.5
Effect Size (Hedges' g)

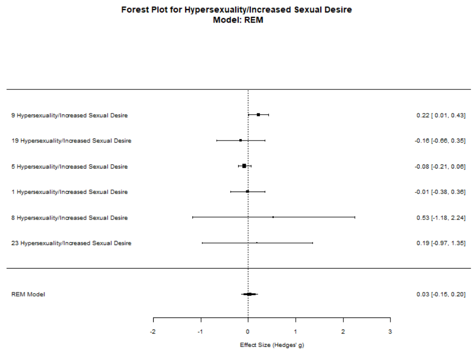
Forest Plot for Hypersexuality/Increased Sexual Desire
Model: REM
9 Hypersexuality/Increased Sexual Desire
0.22 [0.01, 0.43]
19 Hypersexuality/Increased Sexual Desire
-0.16 [-0.66, 0.35]
5 Hypersexuality/Increased Sexual Desire
-0.08 [-0.21, 0.06]
1 Hypersexuality/Increased Sexual Desire
-0.01 [-0.38, 0.36]
8 Hypersexuality/Increased Sexual Desire
0.53 [-1.18, 2.24]
23 Hypersexuality/Increased Sexual Desire
0.19 [-0.97, 1.35]
REM Model
0.03 [-0.15, 0.20]
-2 -1 0 1 2 3
Effect Size (Hedges' g)

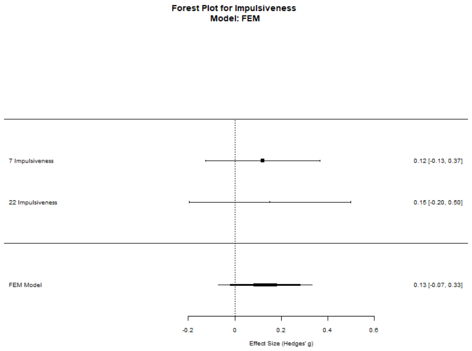

Forest Plot for Impulsiveness
Model: FEM
7 Impulsiveness
22 Impulsiveness
FEM Model
0.12 [-0.13, 0.37]
0.15 [-0.20, 0.50]
0.13 [-0.07, 0.33]
-0.2
0
0.2
0.4
0.6
Effect Size (Hedges' g)

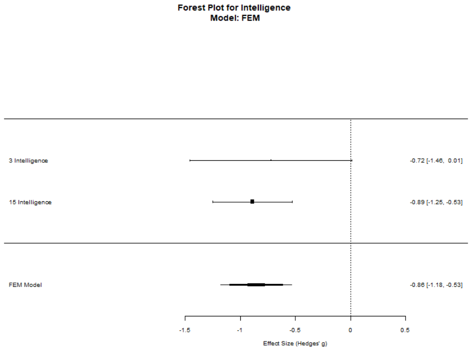

Forest Plot for Intelligence
Model: FEM
3 Intelligence
15 Intelligence
FEM Model
-0.72 [-1.46, 0.01]
-0.89 [-1.25, -0.53]
-0.86 [-1.18, -0.53]
-1.5
-1
-0.5
0
0.5
Effect Size (Hedges' g)

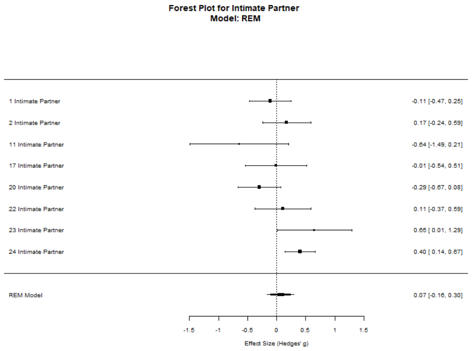

Forest Plot for Intimate Partner
Model: REM
1 Intimate Partner
2 Intimate Partner
11 Intimate Partner
17 Intimate Partner
20 Intimate Partner
22 Intimate Partner
23 Intimate Partner
24 Intimate Partner
REM Model
-0.11 [-0.47, 0.25]
0.17 [-0.24, 0.59]
-0.64 [-1.49, 0.21]
-0.01 [-0.54, 0.51]
-0.29 [-0.67, 0.08]
0.11 [-0.37, 0.59]
0.65 [0.01, 1.29]
0.40 [0.14, 0.67]
0.07 [-0.16, 0.30]
-1.5
-1
-0.5
0
0.5
1
1.5
Effect Size (Hedges' g)

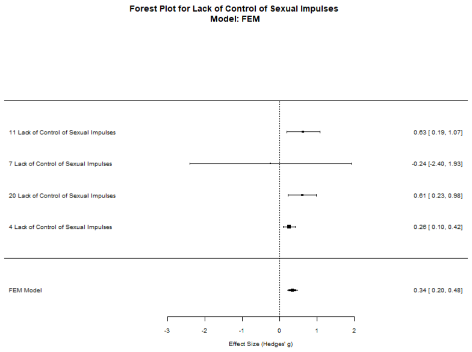

Forest Plot for Lack of Control of Sexual Impulses
Model: FEM
11 Lack of Control of Sexual Impulses
7 Lack of Control of Sexual Impulses
20 Lack of Control of Sexual Impulses
4 Lack of Control of Sexual Impulses
FEM Model
0.63 [0.19, 1.07]
-0.24 [-2.40, 1.93]
0.61 [0.23, 0.98]
0.26 [0.10, 0.42]
0.34 [0.20, 0.48]
-3
-2
-1
0
1
2
Effect Size (Hedges' g)

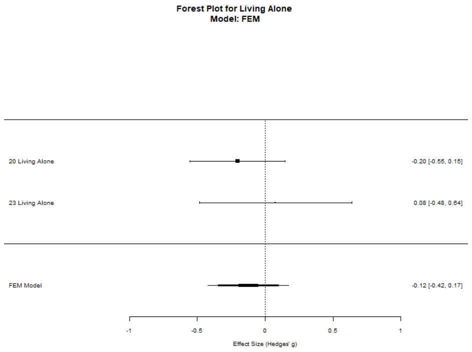

Forest Plot for Living Alone
Model: FEM
20 Living Alone
23 Living Alone
FEM Model
-0.20 [-0.55, 0.15]
0.08 [-0.48, 0.64]
-0.12 [-0.42, 0.17]
-1
-0.5
0
0.5
1
Effect Size (Hedges' g)

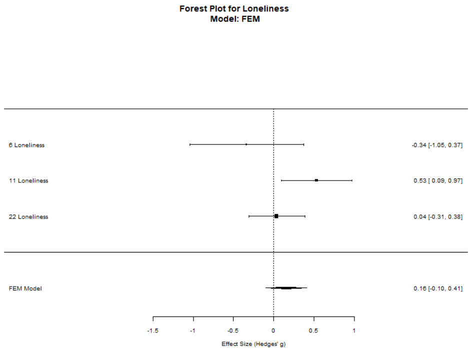

Forest Plot for Loneliness
Model: FEM
6 Loneliness
11 Loneliness
22 Loneliness
FEM Model
-0.34 [-1.05, 0.37]
0.53 [0.09, 0.97]
0.04 [-0.31, 0.38]
0.16 [-0.10, 0.41]
-1.5
-1
-0.5
0
0.5
1
Effect Size (Hedges' g)

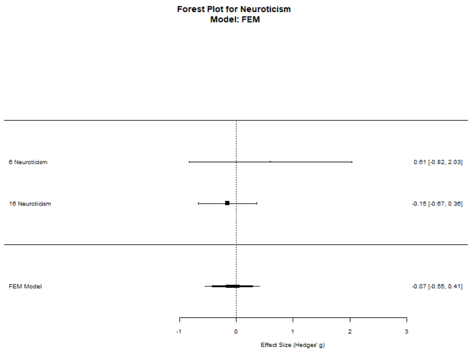

Forest Plot for Neuroticism
Model: FEM
6 Neuroticism
16 Neuroticism
FEM Model
0.61 [-0.82, 2.03]
-0.15 [-0.67, 0.36]
-0.07 [-0.55, 0.41]
-1
0
1
2
3
Effect Size (Hedges' g)

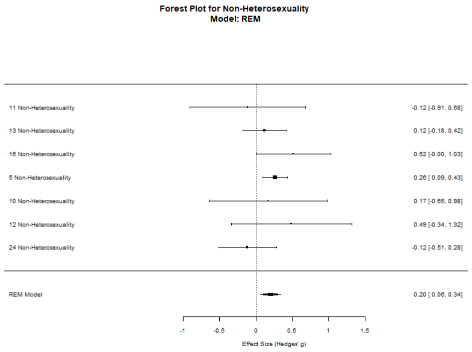

Forest Plot for Non-Heterosexuality
Model: REM
11 Non-Heterosexuality
13 Non-Heterosexuality
16 Non-Heterosexuality
5 Non-Heterosexuality
10 Non-Heterosexuality
12 Non-Heterosexuality
24 Non-Heterosexuality
REM Model
-0.12 [-0.91, 0.68]
0.12 [-0.18, 0.42]
0.52 [-0.00, 1.03]
0.26 [0.09, 0.43]
0.17 [-0.65, 0.98]
0.49 [-0.34, 1.32]
-0.12 [-0.51, 0.28]
0.20 [0.06, 0.34]
-1
-0.5
0
0.5
1
1.5
Effect Size (Hedges' g)

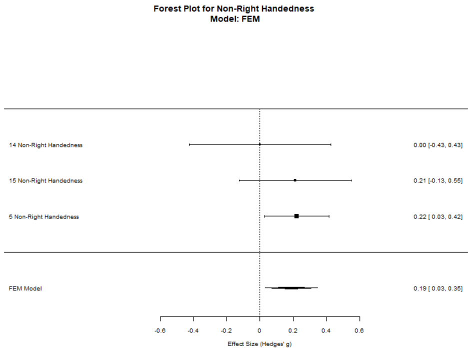

Forest Plot for Non-Right Handedness
Model: FEM
14 Non-Right Handedness
15 Non-Right Handedness
5 Non-Right Handedness
FEM Model
0.00 [-0.43, 0.43]
0.21 [-0.13, 0.55]
0.22 [0.03, 0.42]
0.19 [0.03, 0.35]
-0.6
-0.4
-0.2
0
0.2
0.4
0.6
Effect Size (Hedges' g)

Forest Plot for Obsessive-Compulsive
Model: FEM

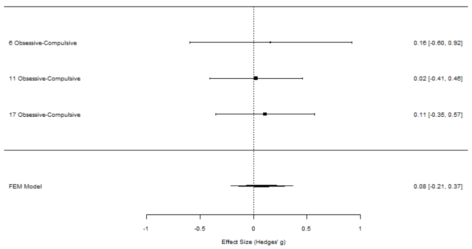

Forest Plot for Openness
Model: FEM

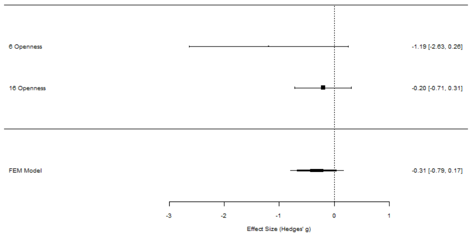

Forest Plot for Other Mental Health Diagnosis
Model: FEM

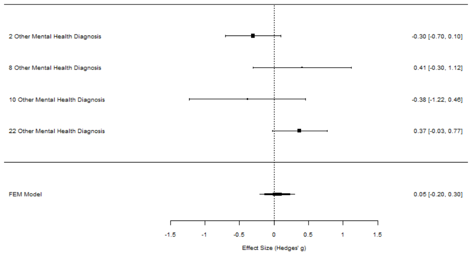

Forest Plot for Own Children
Model: REM

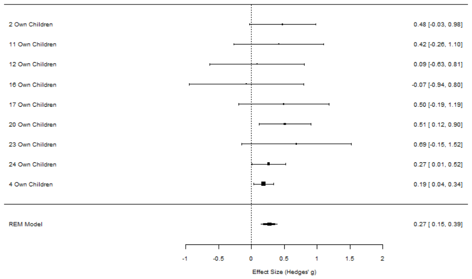

Forest Plot for Paranoid Ideation
Model: FEM

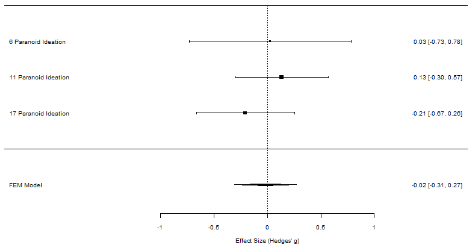

Forest Plot for Phobia
Model: FEM

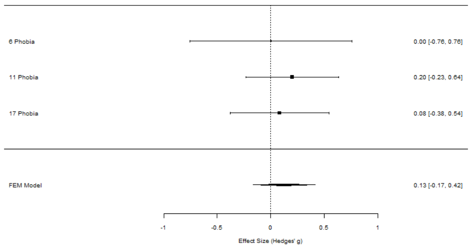

Forest Plot for Preferentiality for Children
Model: REM

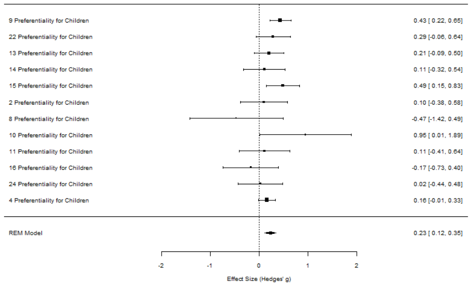

Forest Plot for Preferentiality for Male Children
Model: REM

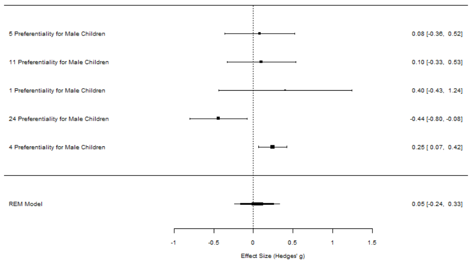

Forest Plot for Psychoticism
Model: FEM

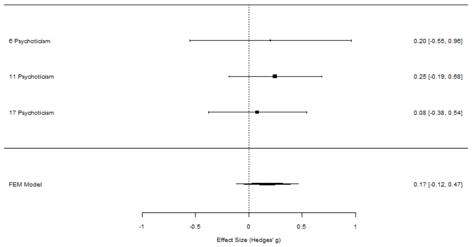

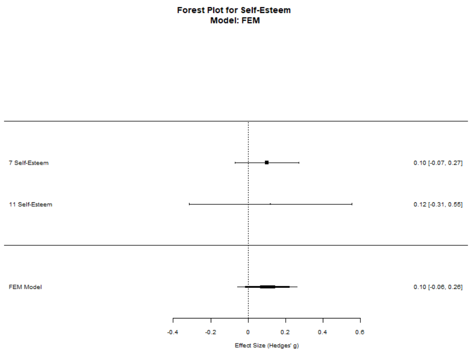
Forest Plot for Self-Esteem
Model: FEM
7 Self-Esteem
0.10 [-0.07, 0.27]
11 Self-Esteem
0.12 [-0.31, 0.55]
FEM Model
0.10 [-0.06, 0.26]
Effect Size (Hedges' g)

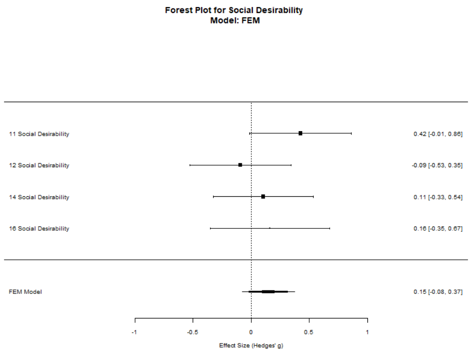
Forest Plot for Social Desirability
Model: FEM
11 Social Desirability
0.42 [-0.01, 0.86]
12 Social Desirability
-0.09 [-0.53, 0.35]
14 Social Desirability
0.11 [-0.33, 0.54]
16 Social Desirability
0.16 [-0.35, 0.67]
FEM Model
0.15 [-0.08, 0.37]
Effect Size (Hedges' g)

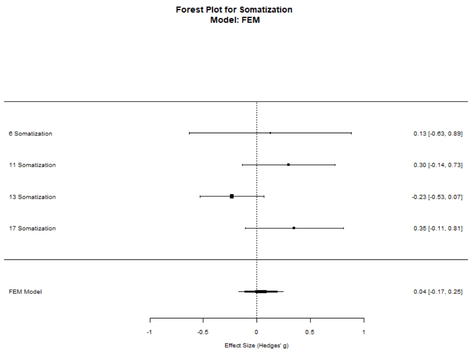
Forest Plot for Somatization
Model: FEM
6 Somatization
0.13 [-0.63, 0.89]
11 Somatization
0.30 [-0.14, 0.73]
13 Somatization
-0.23 [-0.53, 0.07]
17 Somatization
0.35 [-0.11, 0.81]
FEM Model
0.04 [-0.17, 0.25]
Effect Size (Hedges' g)

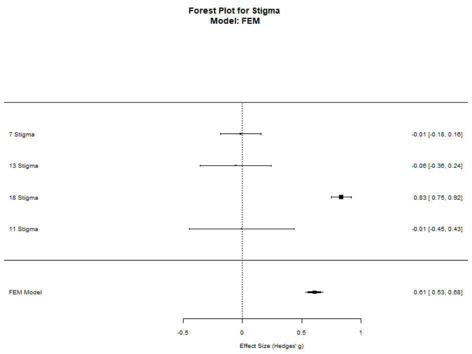
Forest Plot for Stigma
Model: FEM
7 Stigma
-0.01 [-0.18, 0.16]
13 Stigma
-0.06 [-0.36, 0.24]
18 Stigma
0.83 [0.75, 0.92]
11 Stigma
-0.01 [-0.45, 0.43]
FEM Model
0.61 [0.53, 0.68]
Effect Size (Hedges' g)

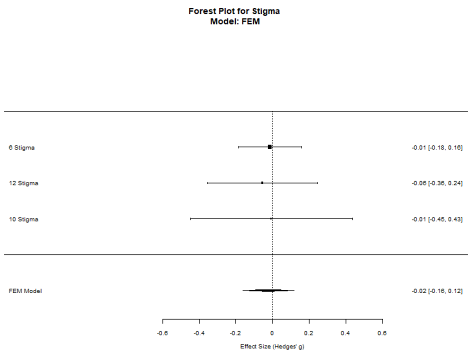
Forest Plot for Stigma
Model: FEM
6 Stigma
-0.01 [-0.18, 0.16]
12 Stigma
-0.06 [-0.36, 0.24]
10 Stigma
-0.01 [-0.45, 0.43]
FEM Model
-0.02 [-0.16, 0.12]
Effect Size (Hedges' g)

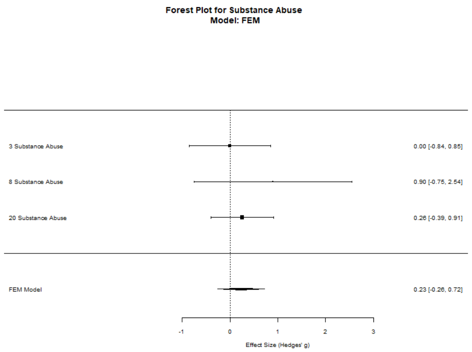
Forest Plot for Substance Abuse
Model: FEM
3 Substance Abuse
0.00 [-0.84, 0.85]
8 Substance Abuse
0.90 [-0.75, 2.54]
20 Substance Abuse
0.26 [-0.39, 0.91]
FEM Model
0.23 [-0.26, 0.72]
Effect Size (Hedges' g)

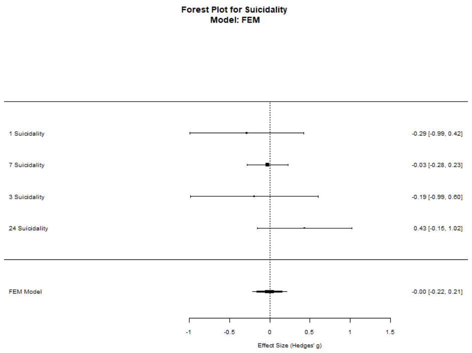
Forest Plot for Suicidality
Model: FEM
1 Suicidality
-0.29 [-0.99, 0.42]
7 Suicidality
-0.03 [-0.28, 0.23]
3 Suicidality
-0.19 [-0.99, 0.60]
24 Suicidality
0.43 [-0.15, 1.02]
FEM Model
-0.00 [-0.22, 0.21]
Effect Size (Hedges' g)

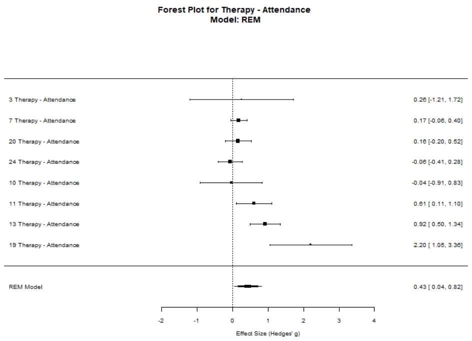
Forest Plot for Therapy - Attendance
Model: REM
3 Therapy - Attendance
0.26 [-1.21, 1.72]
7 Therapy - Attendance
0.17 [-0.06, 0.40]
20 Therapy - Attendance
0.16 [-0.20, 0.52]
24 Therapy - Attendance
-0.06 [-0.41, 0.28]
10 Therapy - Attendance
-0.04 [-0.91, 0.83]
11 Therapy - Attendance
0.61 [0.11, 1.10]
13 Therapy - Attendance
0.92 [0.50, 1.34]
19 Therapy - Attendance
2.20 [1.05, 3.36]
REM Model
0.43 [0.04, 0.82]
Effect Size (Hedges' g)

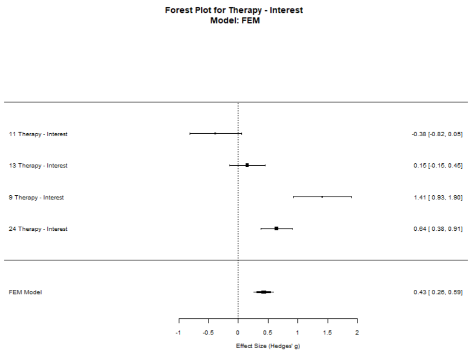
Forest Plot for Therapy - Interest
Model: FEM
11 Therapy - Interest
-0.38 [-0.82, 0.05]
13 Therapy - Interest
0.15 [-0.15, 0.45]
9 Therapy - Interest
1.41 [0.93, 1.90]
24 Therapy - Interest
0.64 [0.38, 0.91]
FEM Model
0.43 [0.26, 0.59]
Effect Size (Hedges' g)

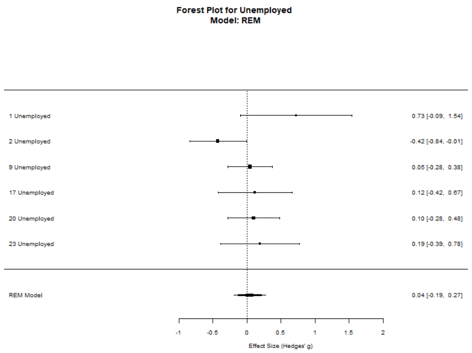
Forest Plot for Unemployed
Model: REM
1 Unemployed
2 Unemployed
9 Unemployed
17 Unemployed
20 Unemployed
23 Unemployed
REM Model
0.73 [-0.09, 1.54]
-0.42 [-0.84, -0.01]
0.05 [-0.28, 0.38]
0.12 [-0.42, 0.67]
0.10 [-0.28, 0.48]
0.19 [-0.39, 0.78]
0.04 [-0.19, 0.27]
-1
-0.5
0
0.5
1
1.5
2
Effect Size (Hedges' g)
